# Machine-Learned Extrapolation of Quantum Mechanical Energies in Implicit Solvent from Short to Long Oligopeptides

**DOI:** 10.1021/acs.jcim.5c02330

**Published:** 2026-04-02

**Authors:** Erik Andris, Ján Michael Kormaník, Tadeáš Kalvoda, Jan Řezáč, Lubomír Rulíšek

**Affiliations:** 89220 Institute of Organic Chemistry and Biochemistry of the Czech Academy of Sciences, Flemingovo náměstí 2, Praha 6 160 00, Czech Republic

## Abstract

Is it possible to obtain a sufficiently accurate quantum mechanical (QM) energy of an arbitrary oligopeptide structure in an *implicit solvent* within a second? Herein, we explore the possibility of constructing potential energy surfaces of larger peptides from rigorous quantum chemical data acquired for hundreds of thousands of capped mono-, di-, and tripeptides. We demonstrate that modern machine-learning methods, in particular NequIP, when trained *only* on tripeptides, can *already* predict QM energies of random decapeptides with a root-mean-square error (RMSE) of <2 kcal mol^–1^. The models also perform well on other out-of-distribution tasks: conformer ranking of a 31-peptide (identifying the global minimum), geometry optimization (RMSD 0.009 Å), and prediction of side-chain interaction energies on PDB structures (with sub-kcal mol^–1^ accuracy). We show that the success of the ML approach is critically dependent on two factors: (i) inclusion of off-equilibrium structures from hot MD sampling, which includes systematic sampling of dihedral angles, and (ii) training on energies of solvated structures, instead of gas-phase energies. Solvated systems are both easier to predict by ML and a more relevant model of typical biomolecular interactions. We make all datasets and models available at doi.org/10.5281/zenodo.15356387.

## Graphical abstract

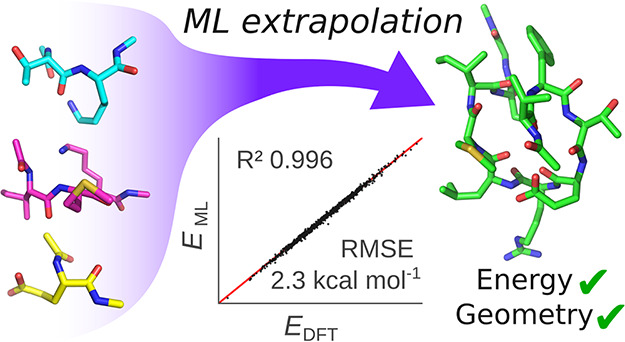

## Introduction

Machine-learning (ML) has recently become an integral part of computational and quantum chemistry.^1–16^ As such, this extremely rapidly developing field has significantly reshaped all of the traditional areas of theoretical chemistry and molecular modeling; be it density functional theory (DFT),^17–23^ wave function theory,^24–26^ classical molecular mechanics or molecular dynamics (MD) simulations,^27–33^ protein–ligand interactions,^34–40^ and many others.^41,42^

At the same time, ML revolutionized protein science and structural biology,^43^ thanks to, e.g., protein structure predictors,^44^ represented by the AlphaFold family of architectures.^45–48^ Somewhere between the two converging fields are our “experimentally-calibrated” computational efforts to predict the structures of peptides, and ultimately proteins, *ab initio* (that is, from the *first principles*).^49,50^ The term *ab initio* here has, as well as in our previous studies, dual meaning. First, it implies the prediction of a peptide/protein structure without any prior knowledge of tens of thousands of experimentally determined crystal structures deposited in the structural databases, such as Protein Data Bank. Second, it denotes the usage of quantum chemistry via DFT or wave function theory in providing accurate and unbiased molecular energies, preferably in solution, for each member of the vast conformational ensembles of oligopeptides. Such an approach has already proven valuable in the construction of empirical force fields.^51,52^ Importantly, we showed that low-energy conformers predicted *in silico* correlate with the structures of their real counterparts in solution, determined experimentally (e.g., by NMR, VCD, and ECD spectroscopies).^50^ However, the necessary conditions for such *ab initio* structure predictions are (i) treatment of solvation effects and (ii) exhaustive conformational sampling. The treatment of solvation effects, which is crucial for modeling biologically relevant systems, has been addressed in ML studies mostly in two ways. This is either via explicit solvation,^53–55^ which is accurate but requires sampling over many system configurations to obtain reliable results.^56,57^ To this end, Moore et al. have recently shown a way to obtain highly accurate solvation energies from a specially trained ML force fields via alchemical free energy calculations.^58^ Another class of methods relies on implicit solvation models, typically employing polarized continuum models (PCM) approaches.^59–64^ The second condition (conformational sampling) is harder to address: exponential growth of the number of unique conformers^65^ with peptide length together with 20*^N^* combinations of amino acids in an *N*-peptide sequence, combined with additional ∼*n* ^2^-*n* ^3^ scaling of practical QM methods, makes these *systematic* efforts computationally prohibitive for *N* > 3. One possibility to advance *ab initio* predictions of peptide/protein structures is to use exhaustive databases of mono-, di-, and tripeptides as “synthetic”^66^ training sets for ML algorithms, specifically modern ML force fields (MLFFs) which have been the subject of intense research efforts in the last years.^67–78^ These ML force fields could predict the QM­(DFT)-level energy of an oligopeptide structure in a fraction of computer time, compared to the “full” (traditional) QM calculation. This might push our ability to generate reliable DFT-quality conformational data for longer peptides, which could in turn be used to train unbiased sequence to structure models for short peptides. However, for this to work, we need models that can extrapolate from short to longer oligopeptides.

Biomolecular models have been, generally, observed to extrapolate quite well. For example, the PhysNet^79^ model, which was trained on combinations of hydrogen-capped fragments (“amons”^80^) of protein structures up to 8 heavy atoms and water molecules to explicitly model solvation effects, is able to predict gas-phase energy and geometry of Ala_10_ peptide with good accuracy. Using explicit solvation is in line with the usual MD approaches, but its high computational cost - mainly the need to sample many solvent configurations - can be limiting for large-scale conformational sampling calculations.

In this work, we explore whether the extrapolation ability will persist when we use isolated polypeptide units (i.e., no dimers) and an implicit solvent model. We demonstrate that this extrapolation is indeed possible and that the extrapolation is facilitated by training on energies of implicitly solvated structures as opposed to the gas-phase energies. These results represent the first step in our ongoing efforts toward more complex tasks, such as *ab initio* protein structure predictions.

## Methods

### Computational Levels

In this work, we used six different computational levels:

i. GFN2 level: GFN2-xTB^81^ semiempirical level with ALPB^82^ solvation model as implemented in the xTB program (version 6.7.0; https://github.com/grimme-lab/xtb).
ii. GFNFF level: GFN-FF^83^ semiempirical level with ALPB solvation model as implemented in the xTB program.
iii. DFT level: BP86 ^84,85^ -D3BJ^86,87^ /dgauss-dzvp^88^ level with special dispersion parameters taken from the literature^89,90^ (α_1_ = 0.7182, s_8_ = 3.2176, α_2_ = 3.8572; denoted as D3BJ^Rezac^) with COSMO^91,92^ solvation model (ε_r_ = 80, FINE cavity^93^) using the TurboMole 7.6, 7.7, and 7.8 programs^94^ (we emphasize that results across these versions do not change).
iv. DFT­(gas) level: BP86-D3BJ^Rezac^/dgauss-dzvp level.
v. COSMO-RS level: identical to DFT level, but the COSMO solvation was calculated at ε_r_ = ∞ and this was followed by the calculation of the COSMO-RS energy.^95^ The final energy is taken as *G* = *E*_COSMO_ + Δ*E* + μ, where *E*_COSMO_ is the COSMO energy at ε_r_ = ∞, ΔE is the averaged correction for the dielectric energy, and μ is the chemical potential of the structure in water. In the calculation, we used BP_TZVPD_FINE_21.ctd parameters supplied with the COSMOTherm 23 (BIOVIA) program.
vi. ff14SBonlysc level: A single-point energy calculation in AMBER 22 ^96^ using force field ff14SBonlysc^97^ and implicit solvent (GBn model, igb = 8) with appropriate atomic radii (mbondi3) as suggested in its Reference Manual.

### Datasets of Random Peptides

To train models capable of predicting peptide energies, we generated datasets of random short peptides from a complete set of 20 natural canonical amino acids. The set contains, in fact, 22 species because it includes the three protonation states of histidine (protonated on Nδ, Nε, and on both, i.e., His^+^). These datasets were created similarly to our previous work (PeptideCS^65^ dataset). The new datasets, which we call PeptideCS2, are available at doi.org/10.5281/zenodo.15356387.

The *training* datasets were conceived and labeled as follows: **T1** comprises capped amino acids (Ace-*X*-NMe), which we also call monopeptides in this work; **T2** are capped dipeptides; and **T3** are capped tripeptides. Examples of these peptides are shown in Figure 1. Each of the datasets contains the *same* number of structures/conformations, arbitrarily chosen as 22^4^ = 234 256 structures, out of which we used 230 000 for training. The number of structures was based on the preliminary training results on the PeptideCS dataset (see Figure S1; note that full PeptideCS comprises >400 million conformers of mono- and dipeptides). Amino acid sequences were sampled evenly during the creation of these datasets, i.e., the number of conformers for each mono-, di-, or tripeptide in each training set, **T1**, **T2,** or **T3**, respectively, was the same. Additionally, a fourth class of datasets, **T123**, which has the same total number of structures as **T1**-**T3** (230 000 for training), was constructed by *random sampling* from **T1**-**T3**, in a “statistical” 1:1:1 mixture (i.e., ∼77 000 structures from each training set appear in **T123**), without stratification by peptide sequence.

**Figure 1:**
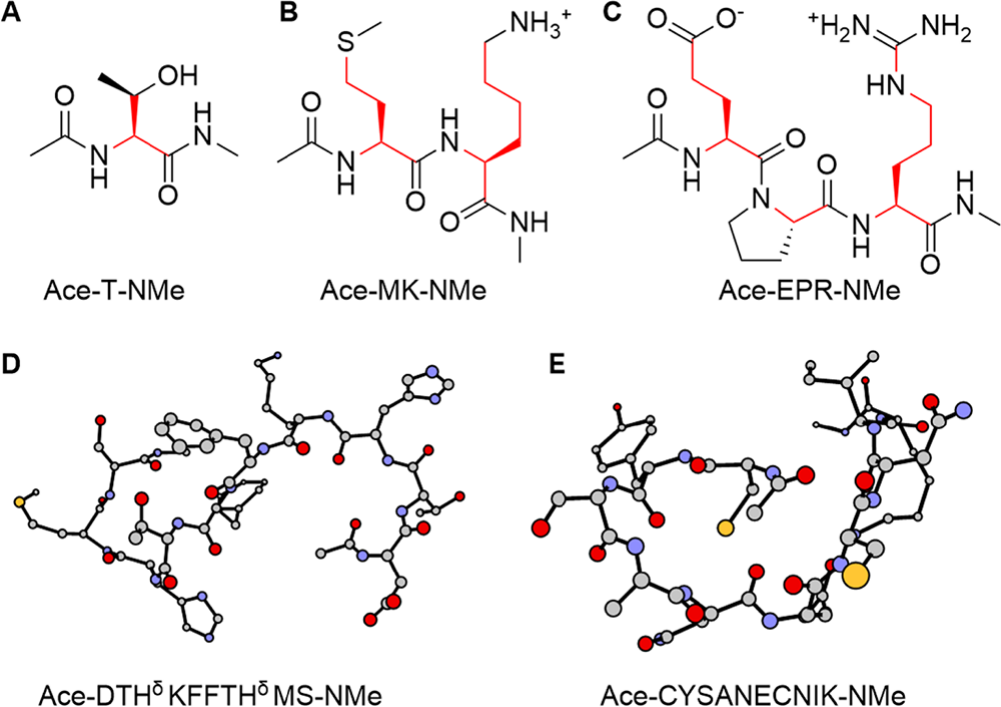
Example peptides from (A) **T1**, (B) **T2**, and (C) **T3** dataset classes. Sampled rotatable bonds are highlighted in red. Example structures from (D) **V10C_H** and (E) **V10CA_H** datasets (*vide infra*). Hydrogens are omitted for clarity.

For validation, we created datasets consisting of (i) 1000 random pentapeptides with 1 conformation per peptide (denoted **V5**), (ii) 1000 random decapeptides with 1 conformation per peptide (**V10**), (iii) 100 conformations of a single randomly selected decapeptide Ace-DTH^δ^KFFTH^δ^MS-NMe (**V10C**, see Figure 1D), (iv) the same with Ace-CYSANECNIK-NMe (**V10CA**, see Figure 1E), (v) 1001 conformers of an uncapped 31-peptide AMH^ε^GATILSVSRFGGDRELLSSQVTQEGEVN generated from MD (**V31C**), and (vi) 803 conformers of uncapped stable miniprotein E6apn1 with known structure (PDB 1RIJ) generated from MD (**V23C**). We tested that energy error estimates differed only negligibly between taking 100 or 1000 structures (Table S1).

Initial structures of all peptides in training and validation sets (except **V31C** and **V23C**) were generated using RDKit.^98^ For each peptide, we also generated a set of random values uniformly sampled from −180° to +180° that were used to constrain all dihedral angles except for the dihedral angle of the peptide bond, ϕ and χ dihedral angles of proline, and dihedral angles of terminal methyl, hydroxyl, amine, and thiol groups. We then minimized each peptide with its constraints first at the GFNFF level, gradually imposing the target dihedral angles by increasing the constraints’ force constants (*c*_F_) in a series of 0.01, 0.02, 0.04, 0.08, and 0.1 hartree, and then at the GFN2 level with force constant of 0.1 hartree (the constraining energy for dihedral angle α is *E*_constrain_ = *c*_F_ (1-cos­(α-α_target_)); as implemented in the xTB program), resulting in the off-equilibrium "**_C**" (**_C** = constrained) dataset. Then, we took these "**_C**" structures and minimized them at the GFN2 level without constraints to yield the "**_M**" (**_M** = minimized) datasets, corresponding to closest local minima. Alternatively, we took the "**_C**" structures and ran a short hot MD (0.3 ps at 800 K, 1 fs time step, using GFN2 level) and took the final frame, which represents a geometry even further from equilibrium. This resulted in the "**_H**" (**_H** = hot) dataset. The naming scheme of the described datasets is shown in Figure 2.

**Figure 2:**
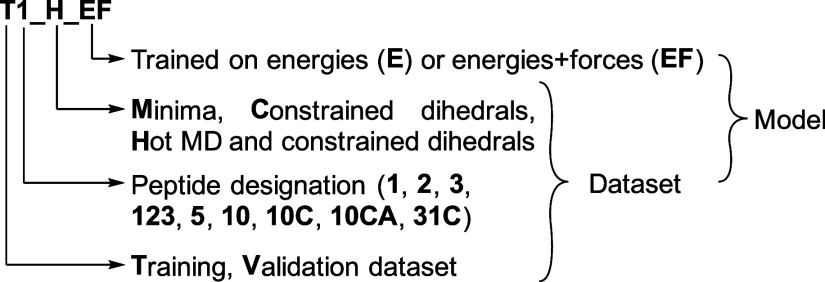
Description of the naming scheme for datasets reported in this work and the models based on these datasets. The dataset names consist of two parts separated by an underscore, such as **V10C_H**. Model names consist of three parts separated by underscores and are derived from their training dataset names by addition of **_E**/**_EF** suffix, such as **T1_H_E** *model* derived from **T1_H** *dataset*.

The energy and energy gradients of all final geometries (**_M**, **_C**, **_H**) were then calculated at the DFT level. This combination (BP86-D3/COSMO//GFN2-xTB) has been previously tested as a sufficiently accurate, yet very economic variant for peptidic structures.^65^ Some of the DFT energy calculations in the **V10**, **V10C**, and **V10CA** datasets failed to converge and the corresponding data points (one to five, out of 100 or 1000) were excluded from further analysis (see Table S2 for the number of data points in each validation dataset). These are typical numbers of DFT “failures” we encounter in studying larger molecular systems (>500 atoms), mostly concerning energetically high-lying conformers.

Additionally, we also recalculated **T1_H**, **T2_H**, **T3_H**, **V5_H**, **V10_H**, **V10C_H**, and **V10CA_H** datasets in the gas phase on the DFT­(gas) level. These datasets are referred to as **T** *x* **(g)_H** and **V** *x* **(g)_H**, respectively.

The **V31C** and **V23C** datasets were constructed as separate datasets of a 31-peptide, AMH^ε^GATILSVSRFGGDRELLSSQVTQEGEVN, and 23-peptide, ALQELLGQWLKDGGPSSGRPPPS, respectively, based on MD simulations performed in Gromacs 2021.4.^99^ For **V23C**, we ran a total of 4 simulations: one from the structure deposited in the PDB and three from random conformers of similar size to the folded structure, but otherwise very different (RMSD between each unfolded structure and the structure from the PDB was 6 Å or higher). The random conformers were prepared by manually editing the dihedral angles of the folded structure to get compact, yet unfolded structures. The simulations were then prepared as follows. Each peptide was put into a cubic box (50 Å, centered around the protein) and solvated with TIP3P^100^ water. AMBER99SB-ILDN^101^ force field was used for all simulations. The water molecules were minimized while the protein was kept restrained. Excess charge was then neutralized and medium ionic strength was added (0.15 M NaCl). The waters and ions were minimized again while the protein was kept restrained. The system was then equilibrated in an NPT ensemble at 310 K and 1 atm with the protein restrained for 1 ns, after which the system was equilibrated again with the same settings, except without the protein restraints. The Berendsen thermostat and barostat were used during the equilibration. The production run was then run for 100 ns using the Nosé-Hoover thermostat at 310 K (**V23C**) or 350 K (**V31C**) and the Parrinello–Rahman barostat at 1 atm. LINCS was used for hydrogen restraints in all simulations. The resulting snapshots (1001 for **V31C**, 804 for **V23C**) were then optimized at GFN-2 level, and had their DFT level single point energies calculated. For **V31C**, only 942 out of 1001 calculations successfully finished and were included in the dataset denoted **V31C_M**. For **V23C**, we selected 201 snapshots from each of the MD simulations. One structure did not finish successfully, resulting in a total of 803 conformers in the dataset denoted **V23C_M**. Due to their construction, these datasets do not have the corresponding **_C** and **_H** datasets.

### APSCI Dataset

To assess interaction between peptide side chains in real proteins, we used structures from the Atlas of Protein Side-Chain Interactions^102^ (APSCI) as used in ref 103. The energy of each side-chain pair was calculated with DFT level without geometry optimization. The side-chain pairs present in APSCI occasionally contain side-chain pairs in which certain side chains contain only the aliphatic chain (e.g., for glutamate, the aliphatic side chain is present without the carboxylic functional group, i.e., only ethane is present instead of propionate). We thus categorized every side-chain pair depending on the charges of the side chains present (e.g., charge of the aforementioned glutamate “side chain” is 0). Histidine is present in APSCI protonated only at Nε (i.e., as H^ε^ in our notation and HIE in the force-field notation) and is always considered as a neutral residue.

### Formation Energies

The energy inputs into the ML models, which we call formation energies *E*_*f*_, were calculated from the DFT energies *E*_DFT_ according to the eqs 1–7, where *q* is molecular charge, *a*, *b*, *c*, *d*, and *e* are stoichiometric coefficients. This was done to remove per-atom contributions, which dominate the total electronic energy, and to ensure that the average energy is close to zero. The energies were likewise expressed in electronvolts (eV) to ensure unit variance.

$$E_{f}\left(C_{a}H_{b}N_{c}O_{d}{S_{e}}^{q}\right)=E_{\mathrm{DFT}}\left(C_{a}H_{b}N_{c}O_{d}{S_{e}}^{q}\right)-aE_{C}-bE_{H}-cE_{N}-dE_{O}-eE_{S}-qE_{q}$$ 1

$$E_{C}=\left(E_{\mathrm{DFT}}\left({\mathrm{CH}}_{4}\right)+E_{\mathrm{DFT}}\left({\mathrm{CO}}_{2}\right)-2E_{\mathrm{DFT}}\left(H_{2}O\right)\right)/2$$ 2

$$E_{H}=\left(E_{\mathrm{DFT}}\left({\mathrm{CH}}_{4}\right)-E_{C}\right)/4$$ 3

$$E_{N}=E_{\mathrm{DFT}}\left({\mathrm{NH}}_{3}\right)-3E_{H}$$ 4

$$E_{O}=E_{\mathrm{DFT}}\left(H_{2}O\right)-2E_{H}$$ 5

$$E_{S}=E_{\mathrm{DFT}}\left(H_{2}S\right)-2E_{H}$$ 6

$$E_{q}=E_{\mathrm{DFT}}\left({{\mathrm{NH}}_{4}}^{+}\right)-E_{N}-4E_{H}$$ 7

Removing the atomic contributions leads to a narrow energy distribution with a width of ∼0.1 hartree (∼60 kcal mol^–1^) for the **T3_C** dataset (other datasets have similar energy distribution based on their construction, shown in Figure S2). This contrasts with ∼1000 hartree distribution of electronic energies when taken directly as inputs for the neural network, or ∼6 hartree if atomization energies were used. This narrower distribution leads to lower prediction errors. The crucial part here is to not only remove the "atomic" contributions, but also to remove the charge contribution, which also leads to a narrower energy distribution (Figure S3). In principle, the same effect could be achieved by least-squares fitting energy of the dataset, but the definitions above are *chemically intuitive* and can be used across different datasets. We note that using *E*_f_ instead of other energies does not change shapes of the potential energy surfaces and merely shifts them closer to zero. Consequently, reaction energy of any reaction (including ionization) expressed in *E*_f_ will be the same as if we expressed it in *E*_DFT_ (Δ_reaction_ *E*_f_ = Δ_reaction_ *E*_DFT_). We also note that the same values of *E*_C/H/N/O/S/q_ were used also to calculate respective *E*_f,COSMO‑RS_ from *E*_COSMO‑RS_ energies.

### Neural Network Training

We trained our models using NequIP,^104^ version 0.6.1 (https://github.com/mir-group/nequip/releases/tag/v0.6.1). The NequIP neural network potentials were trained on a single Nvidia RTX A5000, Nvidia RTX 4090 or Nvidia A100 (40 GB) GPU. We observed that A100 was only about ∼20% faster than RTX4090 and increasing the batch size did not lead to much faster training. Final runs were done on an A100 GPU for 96 h wall clock time. This resulted in different numbers of epochs depending on the training set (monopeptides > dipeptides > tripeptides; see Table S3).

We trained the models on either per-atom MSE of energies (eV; model name suffix **_E**; *n*_molecules_ is the number of molecules in the batch and *n*_atoms_ is the number of atoms in each molecule; eq 8) or the per-atom MSE of energies (eV) and MSE of forces (force = -gradient; eV/Å) with equal importance (model name suffix **_EF**; eq 9).

$$\mathrm{loss}\_E=\left(\Sigma_{\mathrm{molecules}}{\left(\left(E_{f\mathrm{ML}}-E_{f\mathrm{DFT}}\right)/n_{\mathrm{atoms}}\right)}^{2}\right)/n_{\mathrm{molecules}}$$ 8

$$\mathrm{loss}\_\mathrm{EF}=\mathrm{loss}\_E+\left(\Sigma_{\mathrm{molecules}}{\left|{\mathrm{force}}_{\mathrm{ML}}-{\mathrm{force}}_{\mathrm{DFT}}\right|}^{2}\right)/n_{\mathrm{molecules}}$$ 9

Training runs’ validation/training losses can be found in Figures S4–S6 and as separate files in the Supporting Information.

Before training the models, we tested changing the learning rate, number of layers and the number of features, but we did not observe big differences in performance of the resulting models. Because the goal was not to optimize for performance, but rather compare various factors regarding the training sets, we used the same parameter values for all runs: learning rate 0.005, 128 features, 4 layers, batch size 15. Note that these exploratory runs were done using the original PeptideCS dataset,^65^ which is comparable to the **T2_C** dataset used in this paper.

We tested how the neural network potential (NNP) predictions scale with the number of training steps and did not find a validation loss plateau within the allocated time for datasets larger than 200 000 samples (Figure S1). This indicates that overfitting was not a problem for the dataset sizes and numbers of training steps in this work.

### Evaluation Metrics

To evaluate the performance of the trained NequIP models, we found it important to separate the root-mean-square error (RMSE) of the predicted (ML) energies compared to the DFT energies into two components: (i) “shift”, which corresponds to a systematic shift of predicted vs target energies; and (ii) RMSE_shift_, corresponding to the RMSE after the systematic shift has been subtracted. These two numbers and RMSE are related by eq 10.

$$\mathrm{RMSE}={\left({{\mathrm{RMSE}}_{\mathrm{shift}}}^{2}+{\mathrm{shift}}^{2}\right)}^{1/2}$$ 10

Sometimes, especially for tasks such as conformer ranking, we found it useful to include the correlation coefficient of a linear fit of the predicted energies to *target* energies computed at the DFT level, in which we allowed both slope and intercept to be flexible. We refer to it as *R.* ^2^

### Calibration of Neural Network Models for Geometry Optimization

To benchmark the performance of the neural network models in geometry optimizations, we first created a subset of random DFT-optimized structures from **V10_M** dataset using standard DFT geometry optimization tools available in TurboMole program. Next, the structures in this subset were reoptimized to the nearest ML local minima employing the respective ML model using Gaussian 16 ^105^ optimizer (coupled via “external” interface) in Cartesian coordinates.

### Model Training Convergence

The model runs of 96 h did not lead to fully converged models, and we tested longer runs as well. For example, training of the **T3_H_EF** model for additional ∼2 months (we label the resulting model as **T3long_H_EF**) improved all of the predictions of larger peptides (Figure S7). For example, *R* ^2^ on the **V31C_M** dataset improved from 0.98 to 0.99 and errors for decapeptides (full QM energies vs ML-predicted) were close to 1.5 kcal mol^–1^. Moreover, even after hundreds of epochs we did not observe overfitting (Figure S8).

## Results and Discussion

To find out how well a neural network can extrapolate quantum mechanical molecular energies of peptides and proteins in an implicit solvent, we have trained a NequIP^104^ model to predict formation energies *E*_*f*_, defined as the energies required to form the peptides from H_2_O, CO_2_, CH_4_, NH_3_, NH_4_^+^, and H_2_S molecules and their gradients (see Methods). This approach seems, *a posteriori*, to be the key to the accuracy reported below. We trained NequIP on energy gradients of short oligopeptides, up to tripeptides, employing their nonequilibrium geometries which also allowed the resulting models to be used for molecular geometry optimizations.

Briefly, this section is organized as follows: first, we discuss the performance of the NequIP models obtained for the ‘best’ training set(s). The discussion is ordered by the increasing complexity of the validation systems (essentially the length of the peptide chain) and concludes with the application of our models for geometry optimization. Next, we present several important analyses that helped us understand the extrapolation power of our neural network to larger systems: (i) analysis of the performance of our model on the Atlas of Protein Side-Chain Interactions (APSCI) and (ii) analysis of the fundamental differences between the gas-phase predictions (which is a typical domain of most ML approaches for predicting the molecular energies) and our energies in an implicit solvent.

### Model Performance

To systematically evaluate which factors in the training set contribute to the good model performance, we carried out a systematic evaluation of the model quality for *random* pentapeptides and decapeptides. These larger peptides can, in principle, contain all of the interactions present in larger proteins, including interactions between residues which are more distant in sequence and which are, in general, considered important for protein stability.^106^

For practical applications we also need models that can handle off-equilibrium geometries and whose gradients can be used for geometry optimization. To illustrate how the models predict energies on an example, we include predictions on pentapeptides and decapeptides by one of our best models trained on tripeptides, **T3_H_EF**. The performance is depicted in Figure 3. We observe that the RMSE_shift_ for the prediction of the energies of pentapeptides is 1.0 kcal mol^–1^, while the RMSE_shift_ is 2.3 kcal mol^–1^ for the prediction of energies of decapeptides.

**Figure 3:**
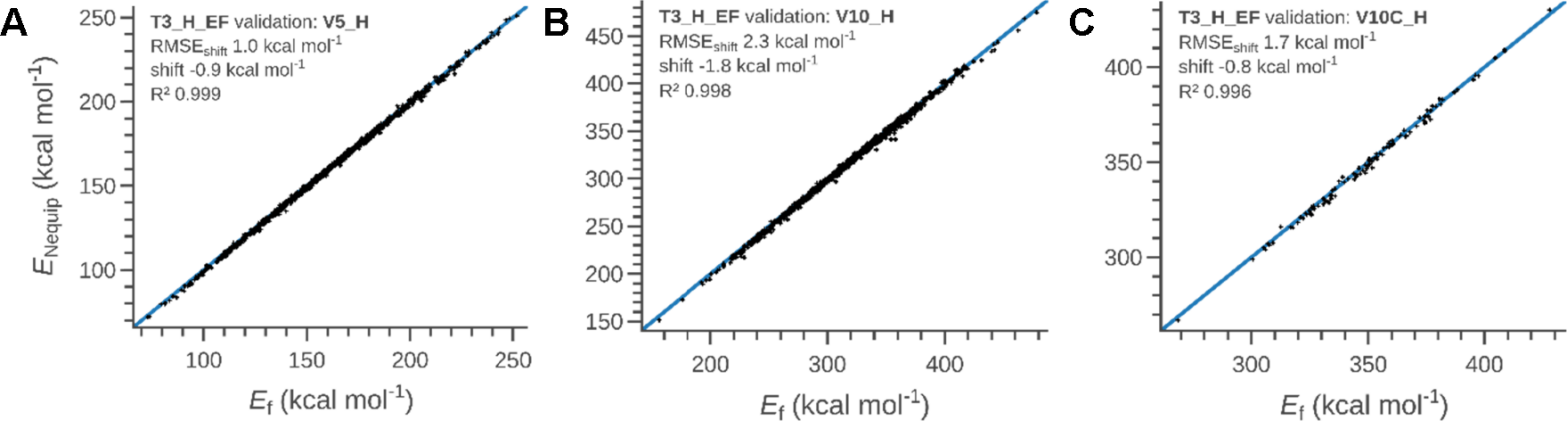
Prediction performance of the **T3_H_EF** model (trained on energies/forces of hot MD structures of constrained tripeptides) on absolute energies of random structures from hot MD of (A) pentapeptides (**V5_H**), (B) decapeptides (**V10_H**), and (C) conformers of single decapeptide (**V10C_H**). The blue line in the graphs represents the function *E*_Nequip_ = *E*_f_.

The trends in RMSE_shift_ of the models were very similar for pentapeptides (**V5**), decapeptides (**V10**), and decapeptide conformers (**V10C**, **V10CA**). In the following discussion, we will thus focus only on the data for **V10** (Figures 4 and 5), while keeping in mind that trends for other validation sets were similar (all results can be found in the Supporting Information as separate image files).

**Figure 4:**
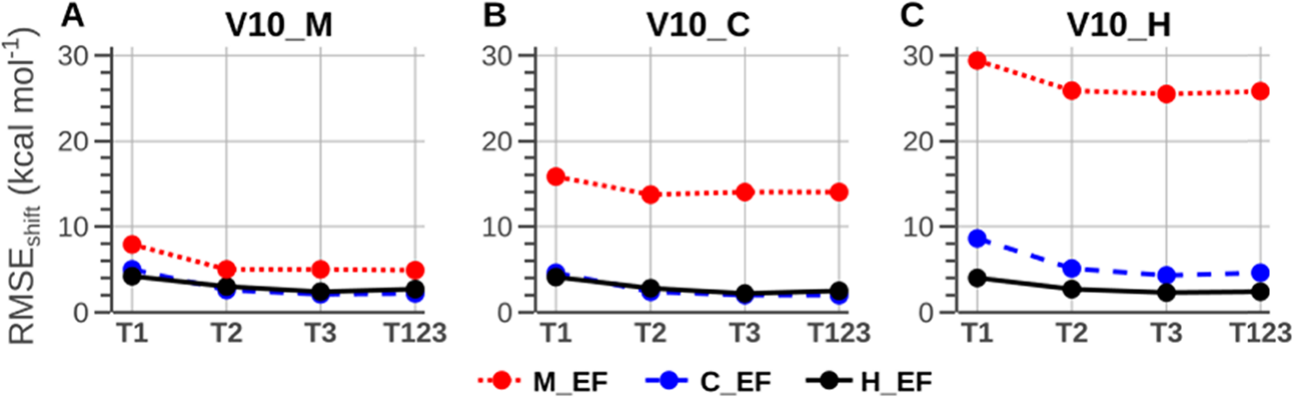
Training dataset dependences of RMSE_shift_ on the peptide length for **V10** datasets: (A) **V10_M**, (B) **V10_C**, (C) **V10_H**. Types of peptides in training sets are distinguished by color/line type: minima (short-dashed red lines), constrained structures (long-dashed blue lines) and structures from constrained hot MD (solid black lines).

**Figure 5:**
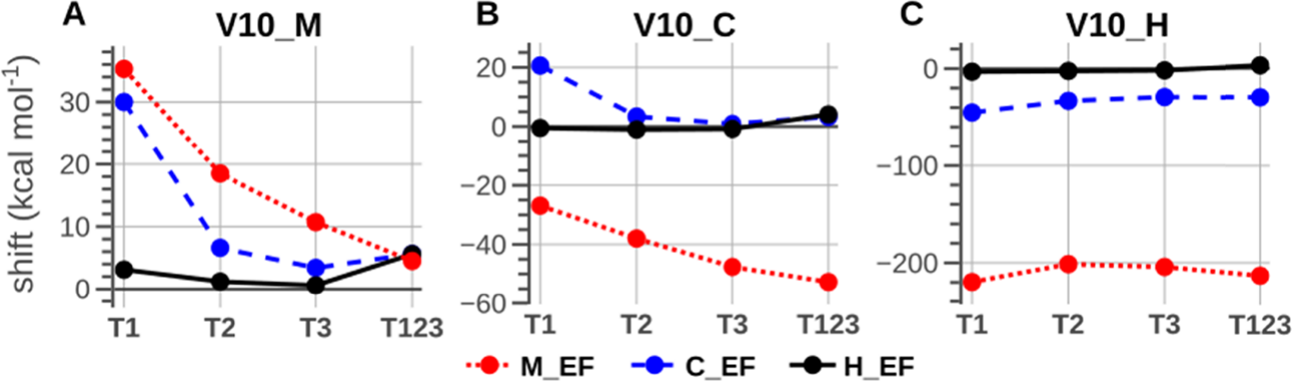
Training dataset dependences of energy shifts on the training peptide lengths for **V10** datasets: (A) **V10_M**, (B) **V10_C**, (C) **V10_H**. Line colors/styles are the same as in Figure 4.

With regard to the length of the peptides in the training set, there is a significant jump in the quality of predictions between models trained only on the monopeptides, i.e., dataset **T1**, and models trained on larger oligopeptides (datasets **T2**, **T3**, and **T123**). This is apparent from all panels in Figure 4. While one would expect that training datasets containing larger peptides (e.g., **T3**) would provide more accurate ML energies of longer oligopeptides, we also wanted to disentangle various types of interactions: **T1** only contains interactions within a given amino acid, whereas **T2** and **T3** also contain pair interactions between amino acid residues. **T3** can, additionally, contain interactions similar to those present in a typical α-helix H-bonding pattern, namely *i*-*i*+4 interactions because the presence of the caps on the *N-* and *C-*termini is just enough to fulfill this condition. However, even the **T1_H_EF** model often shows quite good and somewhat unexpected performance: the RMSE_shift_ across all **V10** datasets is in the range 4.0–4.2 kcal mol^–1^, compared to RMSE_shift_ of **T** *x* **_H_EF** (***x*** = **2**, **3**) models in the range 2.2–3.0 kcal mol^–1^. Figure 4 also shows that performance of the **T123** models is, generally, the same as that of the **T2**-**T3** models. We created the **T123** models to test the effect of size diversity, but this does not improve predictions and we think that diverse lengths of peptides are not very important for training. We will thus not discuss the **T123** models further.

The main factor differentiating the performance of the models is not so much the *size* (**1**, **2**, **3**) of peptides but the *type* (**_M**, **_C** or **_H**) of peptide geometries in the training and validation sets. The local minima (**_M**) represent the simplest baseline, with optimal bond lengths/angles/dihedrals. The **_C** dataset contains structures with distorted dihedral angles. The **_H** dataset contains structures derived from the **_C** dataset by running a short hot MD, which better sample the local energy surface curvature near the **_C** structures. We do not need to sample conformational transitions that happen over longer time periods^107,108^ because such transitions correspond to changes in dihedral angles and these are completely covered by the construction of the **_C** dataset. We observed that inclusion of the off-equilibrium geometries in **_C** and **_H** types of datasets improves predictions in all cases and, generally, **_C** and **_H** type models perform similarly well, with **_H** models having a slight edge in prediction of **_H**-type validation sets only.

We then analyzed the energy *shifts*, which correspond to the global under/overestimation of the true (DFT) energy. Irrespective of the length of the peptide in the validation set, shifts further away from zero are observed if the diversity of the training set (**T** *x* **_M**, **T** *x* **_C**, **T** *x* **_H**) is lower than the diversity in the validation set (where **_M** < **_C** < **_H**). This is perfectly visible in the evaluation in of the **V10_H** dataset in Figure 5C. Models trained on minimum structures (**_M**) show a large negative shift of around −200 kcal mol^–1^ meaning they greatly underestimate the predicted QM energies. The models underestimate the energies because they assume the off-equilibrium structures have a lower energy than they should have, as these models have been trained only on structures with comparatively lower energies. The same is true for models trained on **T** *x* **_C** structures, where the shift is around −40 kcal mol^–1^. Models trained on **T** *x* **_H** structures show shifts between −3.2 (**T1_H_EF**) to −1.8 (**T3_H_EF**) kcal mol^–1^, meaning they predict the *absolute* energies almost exactly. For predictions of **V10_M** and **V10_C**, higher diversity in the training dataset usually moves the *shift* closer to zero, but not monotonically.

### Toward Smaller Proteins: Model Cases of a 31-Residue Peptide and 23-Residue-Folded Miniprotein

Next, we tested the trained models on a practical and at the same time challenging application: the ranking of conformers of a 31-peptide, i.e., the **V31C_M** dataset. The performance of various models and an example energy correlation plot are both shown in Figure 6. Interestingly, most models were able to correctly identify the lowest-energy structure in the dataset, showing the applicability of these models even to much larger peptides. The prediction of absolute energies is also quite good, with the model **T3_H_EF** giving an RMSE_shift_ of 4.7 kcal mol^–1^ and a systematic shift of 3.9 kcal mol^–1^. To put the performance of the model into perspective, we plot correlations between other computational levels and our reference DFT level in Figure S9. The RMSE_shift_ between the “DFT level” and the PBE and TPSS functionals are 3.9 and 1.2 kcal mol^–1^, respectively. Changing the parametrization of D3BJ dispersion had a similar effect, introducing an RMSE_shift_ of 3.1 kcal mol^–1^. Changing the basis set to def2-SVP, however, makes the correlation much worse and gives RMSE_shift_ of 10.8 kcal mol^–1^. Finally, using energies calculated at the GFN2 level results in an RMSE_shift_ of 26.1 kcal mol^–1^ and using energies calculated at the ff14SBonlysc level gives RMSE_shift_ of 18.9 kcal mol^–1^. This shows that using our ML approximation gives errors comparable to changing the DFT functional or basis set. Our evaluations suggest that inclusion of off-equilibrium structures in the training set is the most important consideration – even more important than inclusion of larger peptides (dipeptides and tripeptides). We hypothesize that accurate knowledge of the energy surface of these strained geometries, achieved by their inclusion in training data, is necessary for the success of a particular ML model, because individual residues can adopt strained (off-equilibrium) geometries when they become parts of larger peptides/proteins. It is also clear from the ability of the models to rank conformers of the 31-peptide that the model has acquired at least a rudimentary capacity to describe long-range interactions, i.e., interactions between residues far apart in a peptide sequence. This apparently suffices to capture the important energetic differences between the conformers.

**Figure 6:**
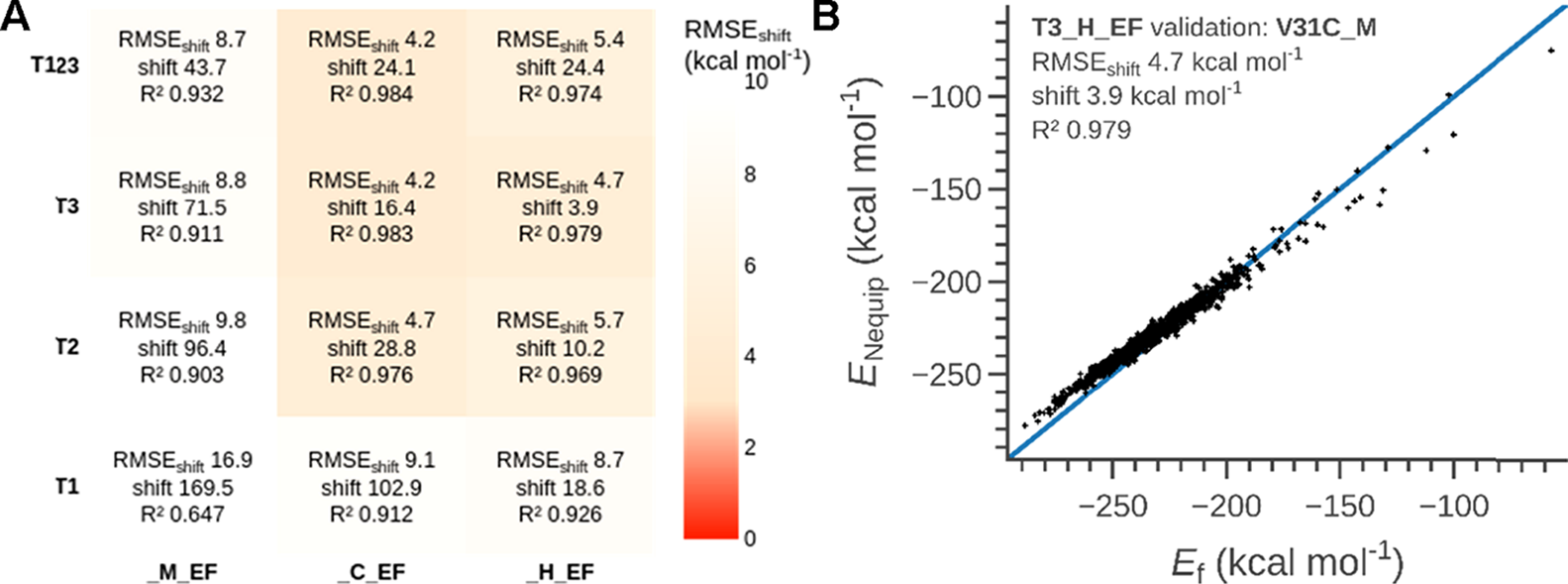
(A) Comparison of performance of various models on the **V31C_M** dataset. (B) Correlation between *E*_f_ and *E*_Nequip_ trained on **T3_H_EF** dataset. Blue line corresponds to *E*_Nequip_ = *E*_f_.

Our results are interesting in the context of a recent study showing the inability of ML models to properly describe these interactions, especially their asymptotic behavior.^109^ Our models can rank the conformers of a 31-peptide even without application of the usual approaches that address the long-range interactions, such as (i) delta-learning, where the model learns just the energy correction from the molecular mechanics (MM)^110^ or semiempirical^111–113^ surfaces to the QM surface, or (ii) inclusion of physics-derived formulas, such as charge and dispersion interactions.^55,114–116^

Despite NequIP being a relatively slow NNFF, its CPU inference time for **V31C** peptide is ∼20 s (the GPU inference takes ∼0.2 s), compared to ∼2 h for the corresponding full DFT calculation. Such a model could thus be easily used to rank conformers of larger peptides, thus reducing the computational time needed by approximately three (CPU) or five (GPU) orders of magnitude. This could lead to a great speed-up in processing of large amounts of data, for example in conformer searches or molecular docking.

Confident with the models’ performance, we further validated the ability of our best model, **T3_H_EF**, to predict the global minimum of a miniprotein with known experimental structure. We’ve selected the miniprotein E6apn1, a Trp-Cage-like miniprotein designed to inhibit the E6 protein of papillomavirus,^117^ whose structure has been determined using NMR (PDB: 1RIJ). This small protein is composed of 23 amino acids, perfectly in the domain of our models, and contains a Trp-Cage-like fold consisting of an α helix and a C-terminal tail folded around a central tryptophan residue. This miniprotein is marginally stable and the unfolded population is quite significant at room temperature (estimated at around 10%). We prepared a validation dataset similar to the **V31C_M** dataset denoted **V23C_M**, in which we included structures from four MD simulations: one run starting from the structure deposited in the PDB (the presumed global minimum) and three runs starting from various unfolded structures similar in size to the global minimum (in all cases, RMSD of the input structure compared to the folded structure was 6 Å or higher). Indeed, the model has ranked structures from the MD simulation of the folded miniprotein as, on average, lower in energy, agreeing well with the experimental observation, despite the marginal stability of this miniprotein (Figure S10). Unlike in the case of **V31C_M**, where we assumed what the minimum is based on the DFT energies, the results for **V23C_M** and conformer ranking also agree with experiment. This confirms that our best model, **T3_H_EF**, despite being trained only on tripeptides, can identify structures close to global minimum even for much larger (mini)­proteins. Considering the fact that the miniprotein E6apn1 was designed as an inhibitor of an oncoprotein, this hints at the potential usefulness of the model within practical applications.

We have also tried running an MD simulation for one of the conformations in the **V23C_M** dataset using the Atomic Simulation Environment library.^118^ Running these simulations would make it possible to both generate structures and obtain their energies simultaneously. During these simulations, the energy was constant within 1 kcal mol^–1^ (Figure S11), confirming that the trained MLFF is of satisfactory quality for MD simulations. However, with a time step of 0.5 fs, typical for *ab initio* MD simulations, we were able to run only 0.2 ns/day on Nvidia RTX 4090 GPU, whereas the simulations ran in GROMACS 2021.4 ran at ∼270 ns/day with explicit solvent. It is thus possible to run the MD simulations using our models, but they are much slower than conventional programs, making e.g. following the folding path of a peptide unfeasible. However, the correlation between energies calculated at the ff14SBonlysc level (see Methods) and the DFT level is much worse (R^2^ 0.71) than the correlation between our model and DFT, opening up a venue to generate conformers through conventional MD and then rank them using our models.

### Geometry Optimization

In the previous evaluations, we compared how the models could approximate DFT energies at the GFN2-xTB-optimized geometries. This is sufficient for our workflows, where we use DFT to rank semiempirical geometries but, ultimately, we would be more interested in using the models also for optimizations, to get DFT-quality geometries as well. To this aim, we optimized 10 random structures from the **V10_M** dataset to the nearest local DFT minima, as described in Methods. We then reoptimized these structures with the trained models to the closest local minimum and compared the energies and geometries with the genuine DFT minima. To evaluate the optimizations, we report two values: (i) Δ*E*_opt_, which is the difference between the energies predicted by the model for the DFT-optimized geometry and the model-optimized geometry, and (ii) RMSD between these two structures. Ideally, both of these values should be zero, as the optimization by the model should not lead to any changes in geometry and thus no change in energy. The results for these optimizations are summarized in Table 1. We observed that the optimization performance was determined more by dataset diversity (where **T** *x* **_M** ≪ **T** *x* **_C** < **T** *x* **_H**) rather than the peptide size. The **T** *x* **_H_EF** combination is required to obtain energy changes below 1 kcal mol^–1^ and corresponding very small geometry RMSDs on the order of 10^–3^ Å, where the structures essentially do not change during optimization with the NNFFs. These results show that the models can be used for practical optimization tasks to obtain geometries corresponding to the DFT level used for their training. Also, it shows that it is not really necessary to include real equilibrium geometries in the training datasets and that geometries optimized at a less accurate level (in this case GFN2-xTB) are sufficient. This is quite important, because optimization to local minima with an expensive method can easily multiply dataset creation costs by a factor of >10 over just taking geometries from a less accurate method.

**Table 1:** Optimization Performance of ML Models^a^

Training set	Δ*E*_opt_(kcal mol^–1^)	RMSD (10^–3^ Å)
**T1_C_EF** ^b^	–47.5	149.7
**T2_C_EF**	–12.4	26.3
**T3_C_EF**	–7.7	29.8
**T123_C_EF**	–9.6	43.2
**T1_H_EF**	–0.7	12.9
**T2_H_EF**	–0.3	9.8
**T3_H_EF**	–0.3	8.5
**T123_H_EF**	–0.3	6.7

a Values correspond to the least accurate out of 10 runs. **T** *x* **_M_EF** models yielded completely broken structures and the optimization did not finish within 300 steps.

b Values calculated out of 7 runs that finished within 300 steps.

### Deciphering the NequIP Performance: Side-Chain Interaction Energies

It came as a surprise to us that ML models trained on short peptides can predict energies of folded structures of *larger* peptides. The latter are dominated by intramolecular interactions between more distant amino acids in the sequence. Indeed, we showed recently that these interactions are even conserved by evolution despite higher backbone strain, rather than low-strained regions.^119^

Thus, as another validation of the models as well as an attempt to understand this surprising behavior of our ML models, we estimated interaction energies of representative side-chain pairs taken from the APSCI dataset, see Methods for details. The results of this validation are shown in Figure 7. In the predictions of absolute energies, our model **T3_H_EF** does very well, reaching RMSE of less than 0.7 kcal mol^–1^ for most groups. The model is able to correctly predict the absolute interaction energies between uncharged groups as well as interactions between uncharged groups and positively charged groups, in both cases reaching RMSE of 0.2 kcal mol^–1^. The model performs worse with interactions between positively charged residues (e.g., Lys-Lys) or interactions involving negatively charged residues (e.g., Asp-Tyr or Asp-Lys). The model performed the worst with interactions between two negatively charged residues (e.g., Asp-Glu).

**Figure 7:**
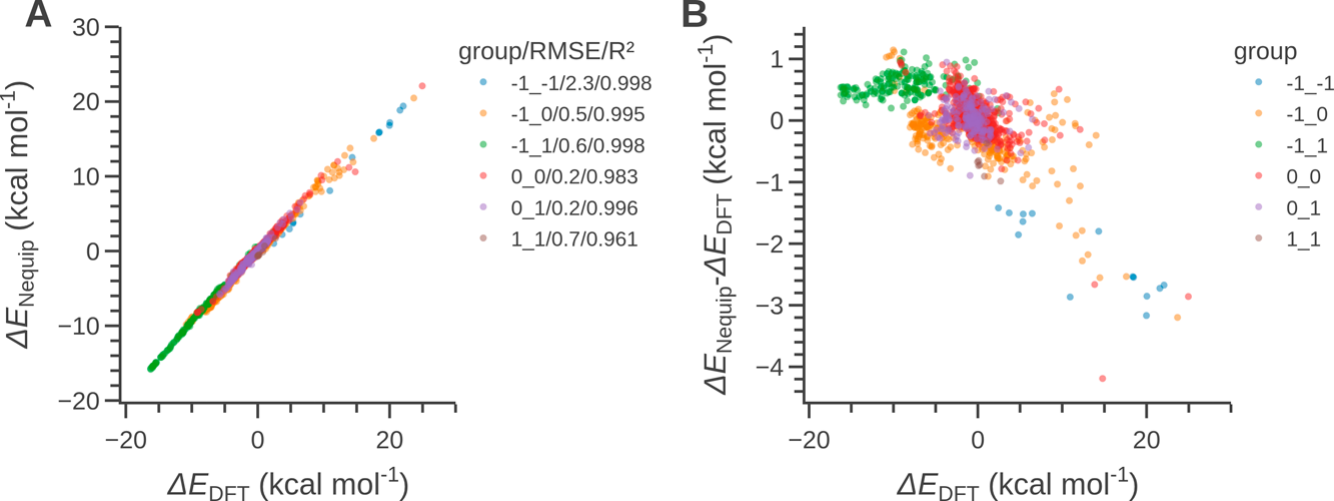
Side-chain pair interaction validation. (A) Correlation between DFT interaction energy Δ*E*_DFT_ and energy calculated by **T3_H_EF**, Δ*E*_Nequip_. (B) Dependence of Δ*E*_Nequip_-Δ*E*_DFT_ on Δ*E*_DFT_. In both graphs Δ*E*_DFT_ and was calculated as *E*_DFT,complex_ – *E*_DFT,fragment1_ – *E*_DFT,fragment2_ and Δ*E*_Nequip_ and was calculated as *E*_Nequip,complex_ – *E*_Nequip,fragment1_ – *E*_Nequip,fragment2._ In both panels (A) and (B), each data point is colored by the charges of the side-chain fragments in the pair, where, e.g., -1_-1 denotes interaction between two negatively charged fragments. RMSE is given in kcal mol^–1^.

We were curious why the model **T3_H_EF** consistently underestimates the energy of repulsive interactions between negatively charged side chains. For example, in some of the structures from the APSCI, the distance between the carboxylate oxygens seems to be shorter than expected in a protein structure. Indeed, these structures are the largest outliers that we observed. Most of the structures with the highest errors are not covered by the training dataset. A similar case is observed in several structures between Tyr and Asp/Glu, in which the phenolic hydrogen of Tyr makes a hydrogen bond with the Asp/Glu. These distances are also quite short and are not covered enough by the training datasets either and might be artifacts in PDB.

We assume that in these repulsive interactions with very short distances, the model **T3_H_EF** is not able to correctly extrapolate what the energy should be because of the steep change of energy with short distances. In order to confirm this hypothesis, we performed scans of pairs involving the acetate anion, the methylammonium cation, and ethane. These showed a large divergence (>5 kcal mol^–1^ in absolute values) between the DFT and **T3_H_EF** energies at distances below 2 Å (Figure S12). This issue is not present with long distances, where the behavior of energy is more predictable. Overall, our model correctly predicts the vast majority of interactions between side chains with minimal errors (errors larger than 1 kcal mol^–1^ are present in <1% of structures).

### Comparison with Standard ML Approaches in Quantum Chemistry: Gas-Phase Energies

In this work, we decided to predict the energies of solvated peptides, because these are the most relevant to the condensed phase. Models trained on gas-phase energies can simulate solvation via explicit solvent molecules, but that approach is very expensive compared to implicit solvent models. However, because most extant ML models predict the energies of molecules in the gas phase, as opposed to solvated energies here, we tested how our protocol would perform for the gas-phase energies. We have thus trained the model **T3­(g)_H_EF**, similar to the **T3_H_EF** model but trained on gas-phase energies. This model performs an order of magnitude worse than the **T3_H_EF** model – the mean absolute error on the internal validation set after 28 epochs (4 days of wall time) was 0.14 eV (3.2 kcal mol^–1^), whereas for the model **T3_H_EF**, the mean absolute error was only 0.02 eV (0.5 kcal mol^–1^; Figure S8).

This is also reflected in the performance on the validation sets: **V10­(g)_H** is predicted by the **T3­(g)_H_EF** model with RMSE_shift_ of 34.1 kcal mol^–1^ as opposed to 2.3 kcal mol^–1^ for **V10_H** by the **T3_H_EF** model. Admittedly, the RMSE_shift_ is lower for the **V10C­(g)_H** dataset at 3.3 kcal mol^–1^, which likely reflects the fact that the total charge of the system is zero (the peptide, however, contains two charged residues).

We note in passing that we also compared the performance of our model with **T3­(g)_H_EF** in predicting relative conformational energies on the **V10C­(g)_H** dataset to that of pretrained models from the literature, namely AIMNet2,^120^ MACE-OFF24­(M),^76^ SO3LR,^55^ and UMA.^121^ We must emphasize that these were trained on energies/gradients calculated using a DFT method different from the **V10C­(g)_H** dataset and the comparison is thus not fair. With that in mind, our model performed similarly to UMA and better than the other models (see Figure S13). These comparisons show that, if we were interested in ranking conformers in the gas phase, we could pick one of the pretrained universal models instead of creating dedicated datasets. However, the energies of peptides in the gas phase and in solution do not correlate well (*R* ^2^ between **V10_H** and **V10­(g)_H** is 0.6, see Figure S14). This limits the direct applicability of the gas-phase models for ranking peptide conformers in solution. Until general-purpose ML models that incorporate implicit solvation become available, using explicit solvation is the only viable “clean” workaround. However, this requires averaging over many solvent configurations, canceling any speedups of the ML algorithms with respect to DFT with implicit solvent. Alternatively, there are semiempirical models available to estimate solvation energies from semiempirical methods,^122^ but these are less accurate.

Part of the relative success of our models comes from predicting solvated energies instead of gas-phase energies. We hypothesize that this is because long-range electrostatic interactions are not as strong in solvated structures because they are attenuated by the solvent, water, due to its high relative permittivity. This enhances the ability of the models to predict the total energy. This result also highlights that we can get more *accurate* and more *relevant* (to biological systems) results if we predict implicitly solvated energies.

### Comparison with Experimental Tripeptide Conformations

To assess the suitability of the NN approximation in “real-world” tasks, we tried to reproduce our previous results obtained while searching for pro-helical and pro-extended tripeptides.^50^ To stay in line with the computational protocol from that study, we trained a new model, **T3­(RS)_H_E**, on the **T3_H** dataset with energies recalculated at the COSMO-RS level. This model, unlike most models mentioned in this work, was trained using only energies and not using energy gradients, because the latter are not accessible at the COSMO-RS level. This is a severe limitation on the amount of training data for the given dataset, because we only get one value per structure, as opposed to 3×(*N*_atoms_)-6 + 1 values (*N*_atoms_ is the number of atoms in the structure) when also using gradients. Despite this limitation, we observed that the results from the **T3­(RS)_H_E** model were in line with the previously experimentally validated COSMO-RS calculations from the work, in the form of secondary structure histograms for selected tripeptides. Namely, the VIV and ALA peptides are predicted to adopt extended conformations (Figures S15 and S16) and EAM is predicted to be a mixture of different conformations (Figure S17). The DIC is predicted to be a mixture of conformations by the **T3­(RS)_H_E** model (Figure S18), but it is the most pro-helical of the tested tripeptides, which was also observed experimentally. A limitation of this validation against experimental data is the fact that the length extrapolation property of the **T3­(RS)_H_E** model has not been tested in this case. When we tried to apply the **T3_H_EF** model to the histogram construction task, the results were less in line with experiments. However, this was expected, because the model was trained using a different DFT method. Training models that will be more accurate with respect to experiments and capable of extrapolation to longer peptides thus remains a goal for our future work.

## Conclusions

We trained the NequIP Neural Network Potential to predict the QM­(DFT-D3)//COSMO energies of larger peptides. Our findings highlight the properties of training datasets that influence whether models trained on shorter peptides extrapolate to longer peptides. We observed significant differences in performance between models trained on monopeptides (**T1**) and longer peptides (**T2**, **T3**), confirming that pair-residue interactions are, as expected, vital in the protein context and proteins cannot be viewed as sequences of independent amino acids. Thus, the minimal training set is represented by dipeptides, with additional (smaller) improvement obtained for tripeptides (**T3**). Even more important, however, was the inclusion of constrained structures from 800 K (hot) MD (**_H**) in the training data. Our best model **T3_H_EF** achieved an excellent *R* ^2^ of 0.998 and RMSE_shift_ of 2.3 kcal mol^–1^ on the decapeptide validation set **V10_H**. We also showed that the model is able to correctly rank conformers of a 31-peptide (*R* ^2^ 0.979; RMSE_shift_ of 4.7 kcal mol^–1^) and discern the global minimum structure of a stable miniprotein from nonfolded structures, illustrating its generalization and extrapolation abilities within the constrained chemical space of peptides. These are also responsible for the sub-kcal mol^–1^ accuracy on the set of side-chain interaction energies from the APSCI. Furthermore, the model can be used for geometry optimizations, drastically reducing the required computational time compared to DFT (by approximately 3–5 orders of magnitude), as well as MD simulations, even though they’re much slower (by >3 orders of magnitude) than simulations using MM force fields. We speculate that the generalization to longer peptides is possible due to the prediction of energies in implicit solvent, which dampens long-range electrostatic interactions and which we found to be easier to predict than gas-phase energies. Moreover, predicting molecular structures and properties in implicit solvent is much more relevant to biochemistry, making our presented results of both conceptual and practical interest. A potential limitation is the inability of the presented models to describe high-energy parts of the potential energy surface, caused by the lack of such structures in the training data. This could limit their use in MD settings. However, we still believe that finding and accurately characterizing potential energy surfaces of peptides of increasing complexity (length) without the need for time-consuming QM calculations may enable the *ab initio* protein structure prediction. A ‘**T2/T3** → **V** *n*’ approach used here (i.e., predicting accurate QM energies of longer folded peptides from dipeptides or tripeptides) could point to the existence of a set of rules underlying *ab initio* protein folding, further confirming our hypothesis of relatively short folding seeds^50^ in protein structures.

## Supplementary Material



## Data Availability

Input files and python libraries, including custom dataloader for our datasets, which are necessary to reproduce the training runs, final model weights for the NequIP 0.6.1 models, notebook for model inference, and images of training runs and model performance are available on doi.org/10.5281/zenodo.15356387.

## Notes

Performance of trained models on subsets of **V10_C** dataset (Table S1), number of structures in validation datasets (Table S2), and number of training epochs (Table S3); validation loss as a function of dataset size (Figure S1); energy histograms of **T3** training datasets (Figures S2 andS3); hyperparameter scans (Figures S4–S6); **T3_H_EF** and **T3long_H_EF** model accuracy (Figure S7); comparison of training on gas-phase and solvated energies (Figure S8); comparison of conformational energies in the **V31C_M** dataset at different levels (Figure S9); MD simulations of E6apn1 miniprotein (Figures S10 and S11); interaction energies of CH_3_COO^–^, MeNH_3_^+^, and C_2_H_6_ pairs (Figure S12); prediction of conformational energies of the **V10C­(g)_H** dataset by pretrained NNFFs (Figure S13); energy correlation between the **V10C_H** and **V10C­(g)_H** datasets (Figure S14); and energy histograms of ALA, VIV, EAM, and DIC tripeptide conformers from ref 50 (Figures S15–S18) (PDF)
