# Supplementary material for: Machine-Learned Extrapolation of Quantum Mechanical Energies in Implicit Solvent from Short to Long Oligopeptides
Source: J Chem Inf Model. 2026 Apr 2;66(11):6531–43. doi: 10.1021/acs.jcim.5c02330 (PMC13250900; doi:10.1021/acs.jcim.5c02330)
Supplement: Supplementary file 1 [file ci5c02330_si_001.pdf]

Supporting Information

Machine-Learned Extrapolation of Quantum Mechanical Energies in Implicit Solvent from Short to Long Oligopeptides

Erik Andris,* Ján Michael Kormaník, Tadeáš Kalvoda, Jan Řezáč, Lubomír Rulíšek*
Institute of Organic Chemistry and Biochemistry of the Czech Academy of Sciences, Flemingovo náměstí 2, 160 00 Praha 6, Czech Republic.

Input files and python libraries, including custom dataloader for our datasets, which are necessary to reproduce the training runs, final model weights for the NequIP 0.6.1 models, notebook for model inference, and images of training runs and model performance are available on doi.org/10.5281/zenodo.15356387.

Table S1. Performance of trained models evaluated on **V10_C** dataset (995 data points) and its subsets (100 data points each). Subset 1 comprises every 10th structure. Subset 2 comprises the first 100 structures.

Model	$RMSE_{shift}$ (kcal mol^{-1})		
	Full dataset	Subset 1	Subset 2
**T123_C_E**	2.3	2.6	2.0
**T123_C_EF**	2.0	2.1	1.6
**T123_M_E**	8.2	7.5	6.8
**T123_M_EF**	14.0	11.6	13.0
**T123_H_E**	2.3	2.6	2.0
**T123_H_EF**	2.5	2.5	2.0
**T1_C_E**	7.7	8.0	7.2
**T1_C_EF**	4.6	4.2	4.3
**T1_M_E**	16.8	17.1	15.5
**T1_M_EF**	15.8	13.7	14.4
**T1_H_E**	4.7	5.4	4.0
**T1_H_EF**	4.1	4.1	3.7
**T2_C_E**	2.6	2.6	1.9
**T2_C_EF**	2.4	2.3	2.0
**T2_M_E**	9.8	9.0	8.9
**T2_M_EF**	13.7	11.6	12.7
**T2_H_E**	2.6	2.8	2.1
**T2_H_EF**	2.8	2.8	2.3
**T3_C_E**	2.0	2.3	1.7
**T3_C_EF**	2.0	1.9	1.5
**T3_M_E**	7.1	6.5	6.0
**T3_M_EF**	14.0	11.8	13.0
**T3_H_E**	2.2	2.4	1.8
**T3_H_EF**	2.2	2.3	1.8

Table S2. Number of structures in validation datasets.

Dataset	Number of structures
**V5_C**	1000
**V5_M**	1000
**V5_H**	1000
**V10_C**	995
**V10_M**	996
**V10_H**	996
**V10C_C**	99
**V10C_M**	99
**V10C_H**	99
**V10CA_C**	99
**V10CA_M**	99
**V10CA_H**	99
**V31C_M**	942

Table S3. Number of epochs for different training runs.

Dataset	Epochs
**T123_C_E**	76
**T123_C_EF**	40
**T123_M_E**	76
**T123_M_EF**	40
**T123_H_E**	76
**T123_H_EF**	40
**T1_C_E**	128
**T1_C_EF**	72
**T1_M_E**	128
**T1_M_EF**	70
**T1_H_E**	129
**T1_H_EF**	72
**T2_C_E**	79
**T2_C_EF**	41
**T2_M_E**	76
**T2_M_EF**	40
**T2_H_E**	79
**T2_H_EF**	44
**T3_C_E**	56
**T3_C_EF**	28
**T3_M_E**	51
**T3_M_EF**	28
**T3_H_E**	56
**T3_H_EF**	28
**T3long_H_EF**	483
**T3(RS)_H_E**	57
**T3(g)_H_EF**	28

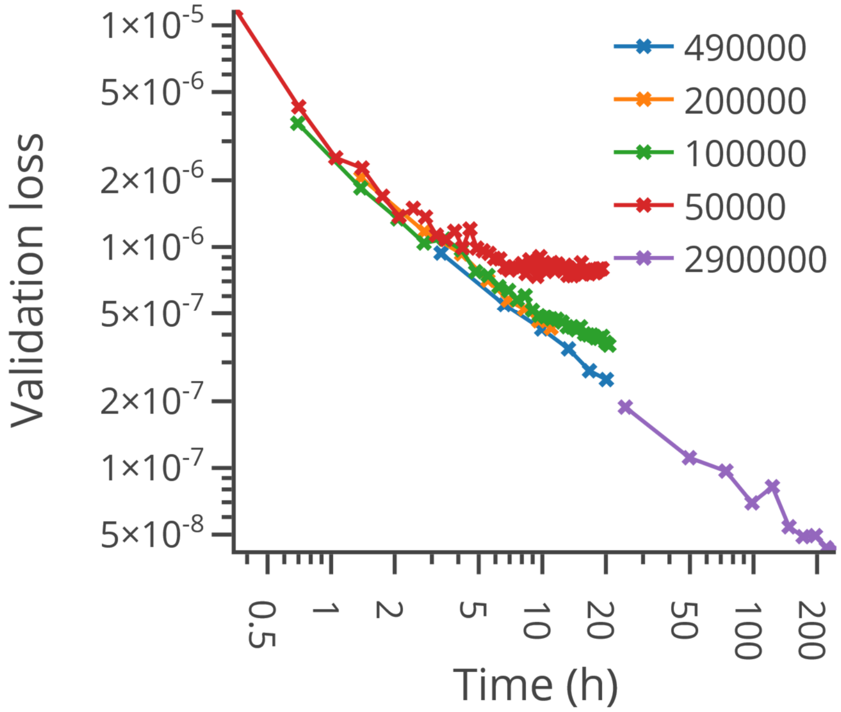

Figure S1. Validation RMSE energy on PeptideCS dataset as a function of training set size.

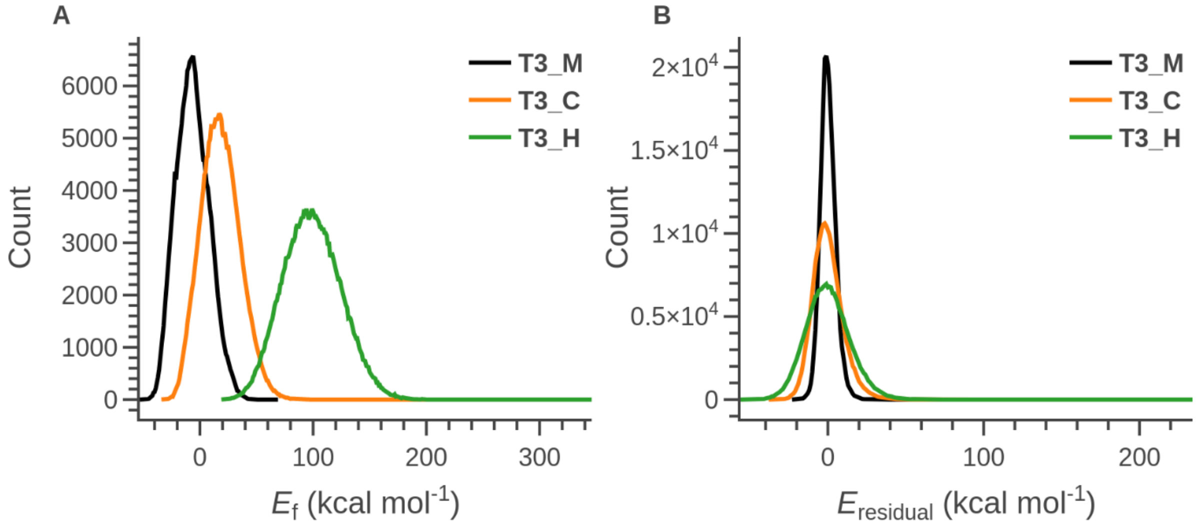

Figure S2. Energy histograms of structures in different **T3** training datasets. (A) E_f energies, (B) Residual energies, after removing contributions to E_f by amino acid residues ($E_{residual} = E_f - E_{residue1} - E_{residue2} - E_{residue3}$). Energies of residues depend only on the amino acid identity (not its position within sequence) and were calculated to minimize the sum of $|E_{residual}|^2$ across each dataset.

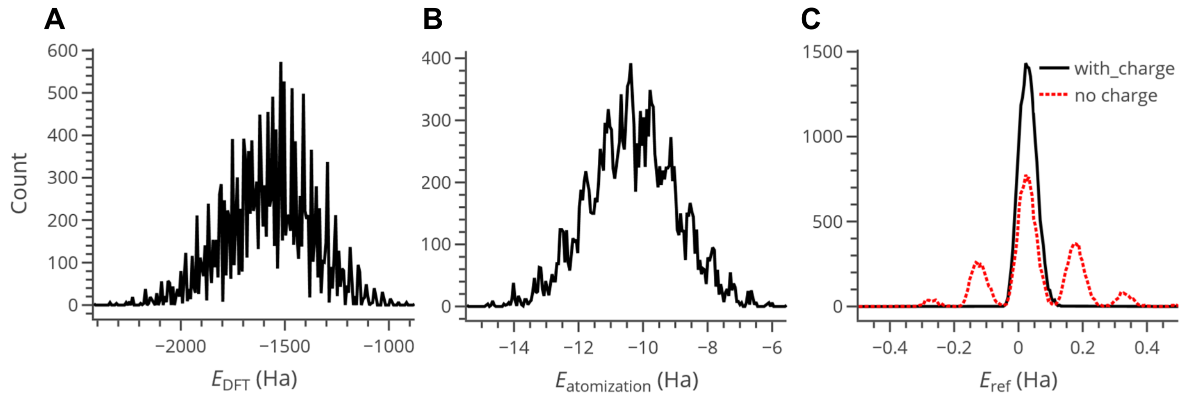

Figure S3. Energy distributions of the **T3_C** dataset. (A) Electronic energies. (B) Atomization energies. (C) Formation energies without inclusion of charge (red dashed line) and with inclusion of charge (solid black line).

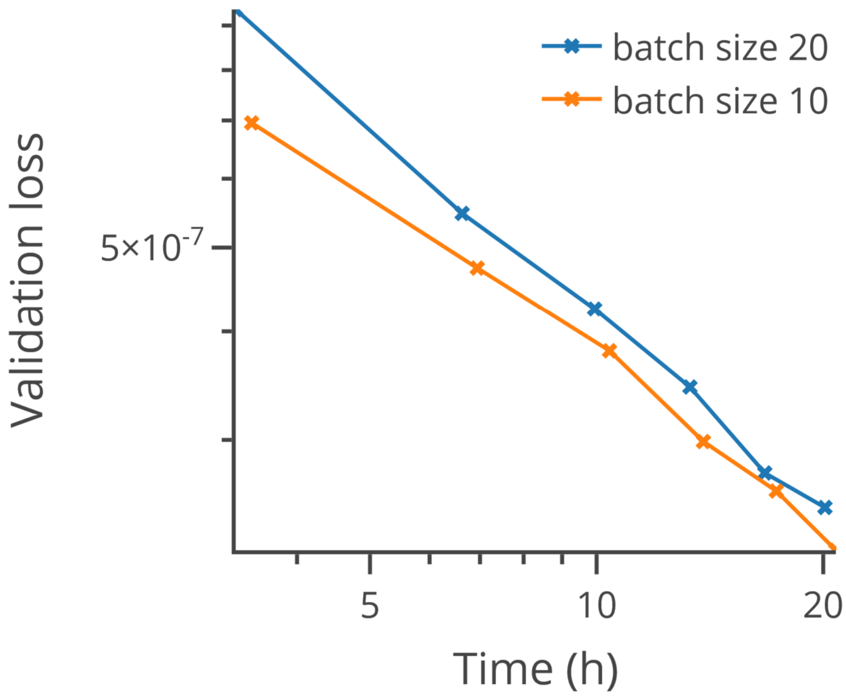

Figure S4. Training curves with different batch sizes. The crosses indicate individual epochs.

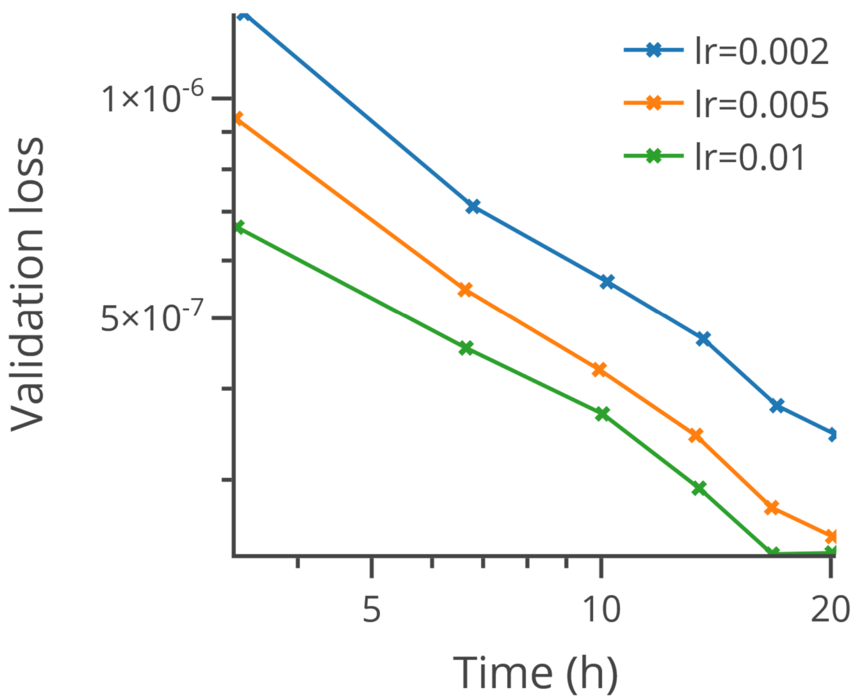

Figure S5. Validation loss as a function of learning rate (lr). The crosses indicate individual epochs.

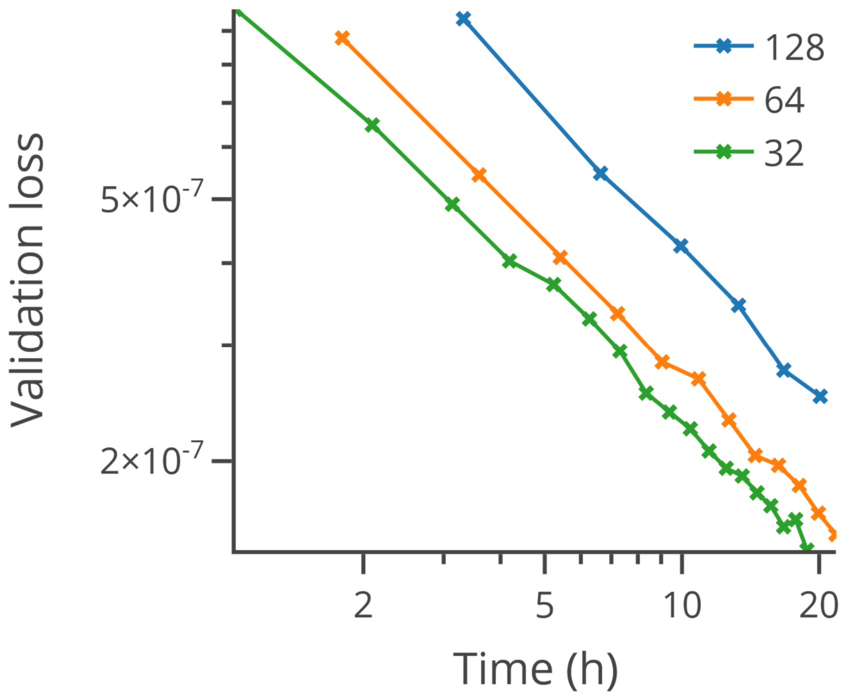

Figure S6. Validation loss as a function of the number of features. The crosses indicate individual epochs.

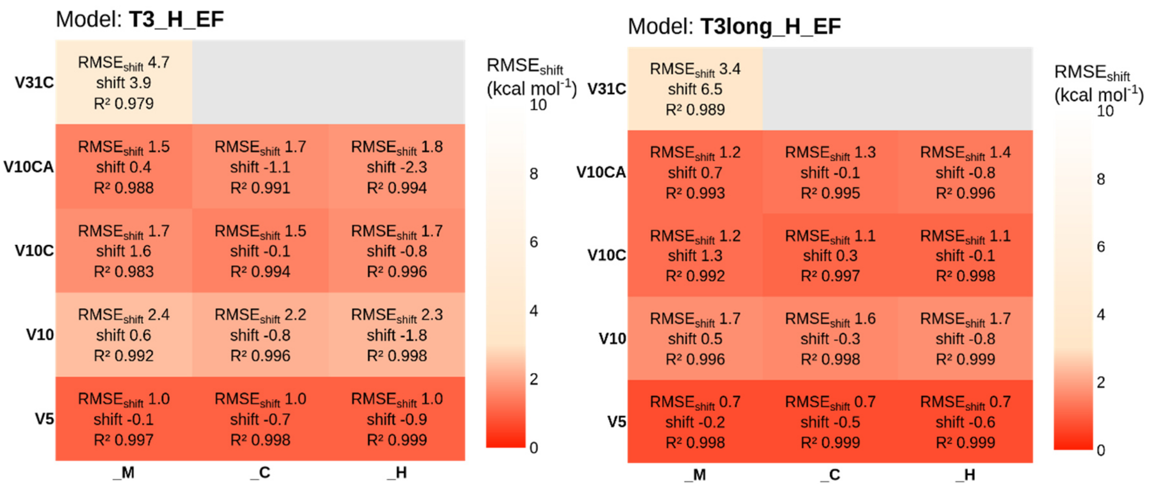

Figure S7. Accuracy of **T3_H_EF** model with the standard training of 4 days (left) and with the longer training (model **T3long_H_EF**, >2 months). We observe improvement in all correlations, especially on the **V31C_M** dataset.

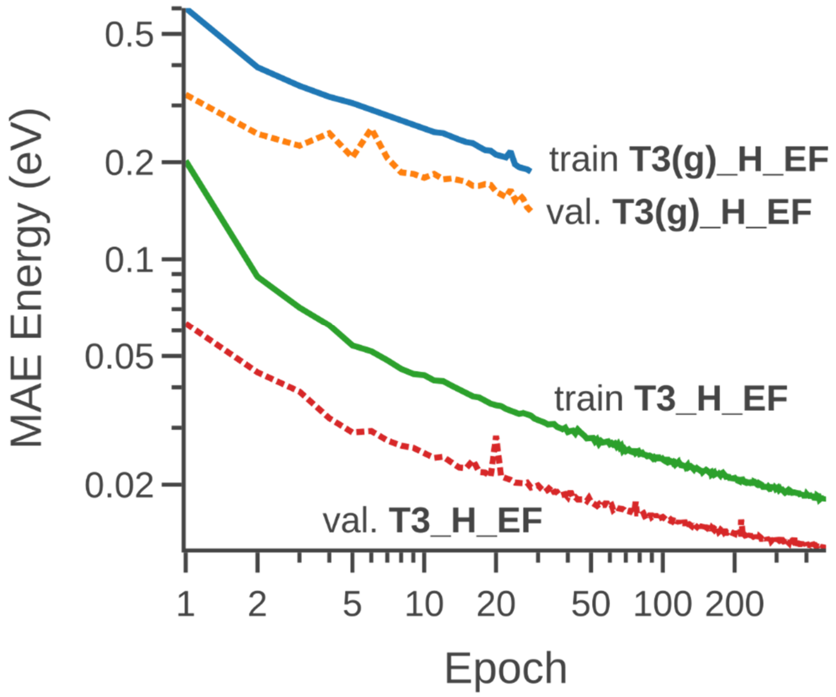

Figure S8. Dependence of mean absolute error in E_f for training and validation sets for **T3_H_EF** dataset on epoch number for model trained/evaluated on gas-phase energies (blue and dashed orange line) and model trained on energies in solution (green and dashed red line).

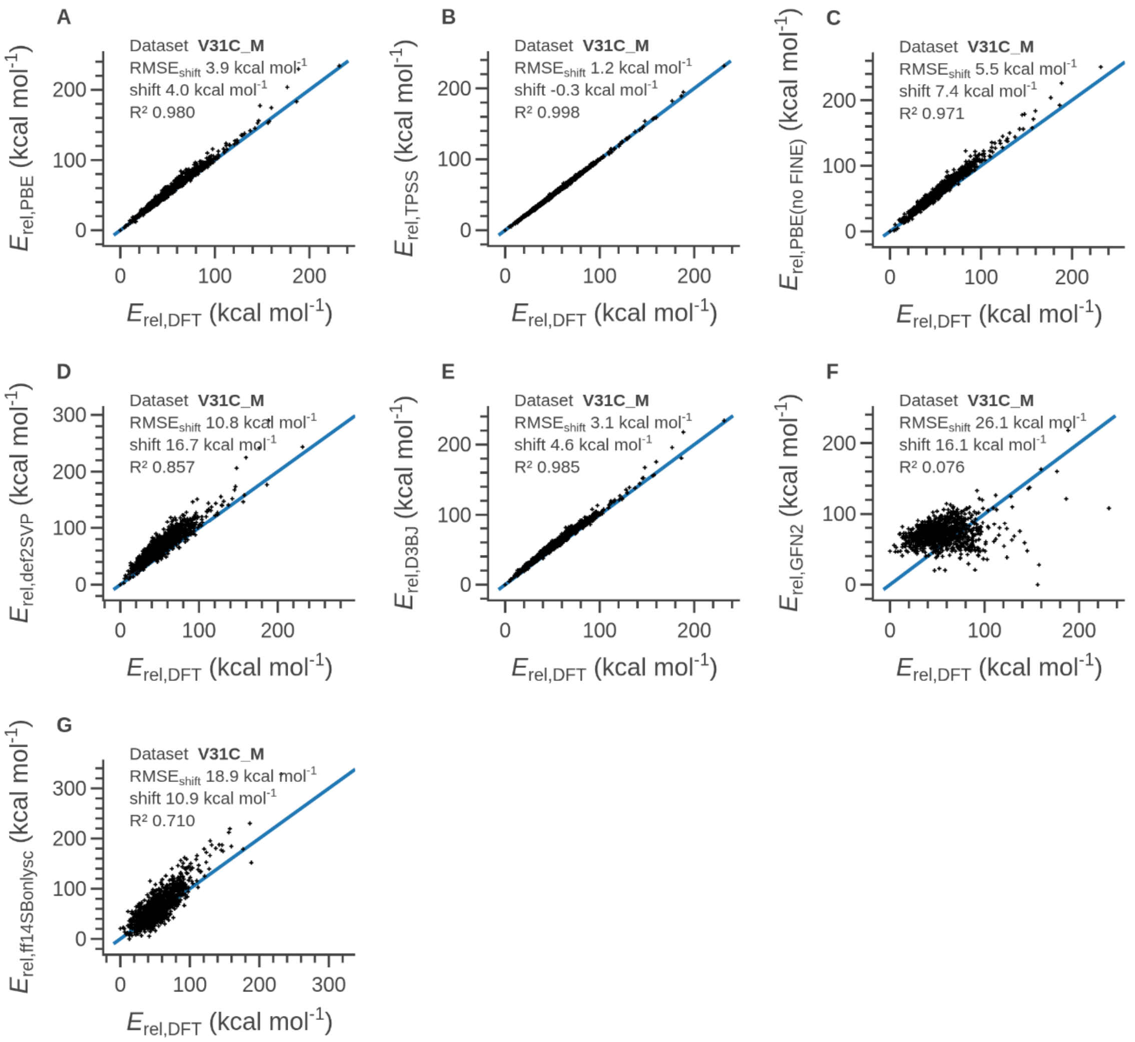

Figure S9. Correlation between relative conformational energies at the DFT level ($E_{\mathrm{rel,DFT}}$) on **V31C_M** dataset and relative conformational energies at (A) DFT level with PBE functional (all other parameters were kept the same), (B) DFT level with TPSS functional (all other parameters were kept the same), (C) DFT level with PBE functional and "normal" COSMO cavity (not FINE cavity; all other parameters were kept the same), (D) DFT level with def2-SVP basis (all other parameters were kept the same), (E) DFT level with default D3BJ dispersion parameters (all other parameters were kept the same), (F) GFN2 level, and (G) ff14SBonlysc level. Only 931 out of 942 structures, which were successfully calculated at all levels, were included in these correlations.

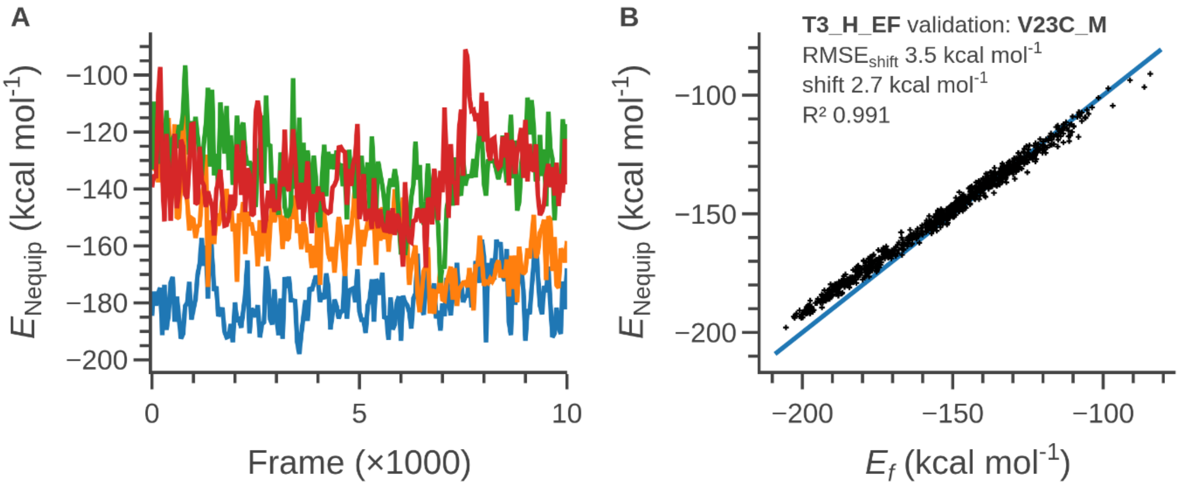

Figure S10. (A) **T3_H_EF** energies of GFN2-level minimized snapshots from MD simulations of the protein E6apn1 (PDB: 1RIJ), starting from folded structure (blue) and three unfolded structures (red, green, orange). (B) Correlation between the DFT-calculated E_f of the MD snapshots (collectively labeled as dataset **V23C_M**) and the E_f energies predicted by **T3_H_EF** model.

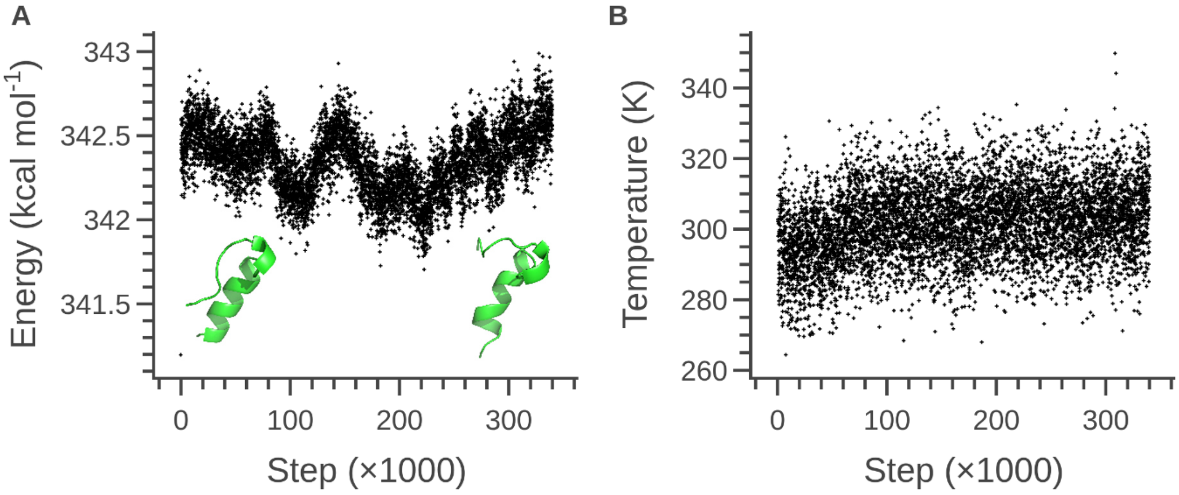

Figure S11. (A) Total energy and (B) temperature during NVE MD simulation of E6apn1 (PDB: 1RIJ) with **T3_H_EF** model. During the simulation, we used velocity Verlet dynamics with 0.5 fs timestep and initial kinetic energy drawn from Maxwell-Boltzmann distribution at 530 K. The structures in panel (A) correspond to the first (left) and the last frame (right) of the simulation.

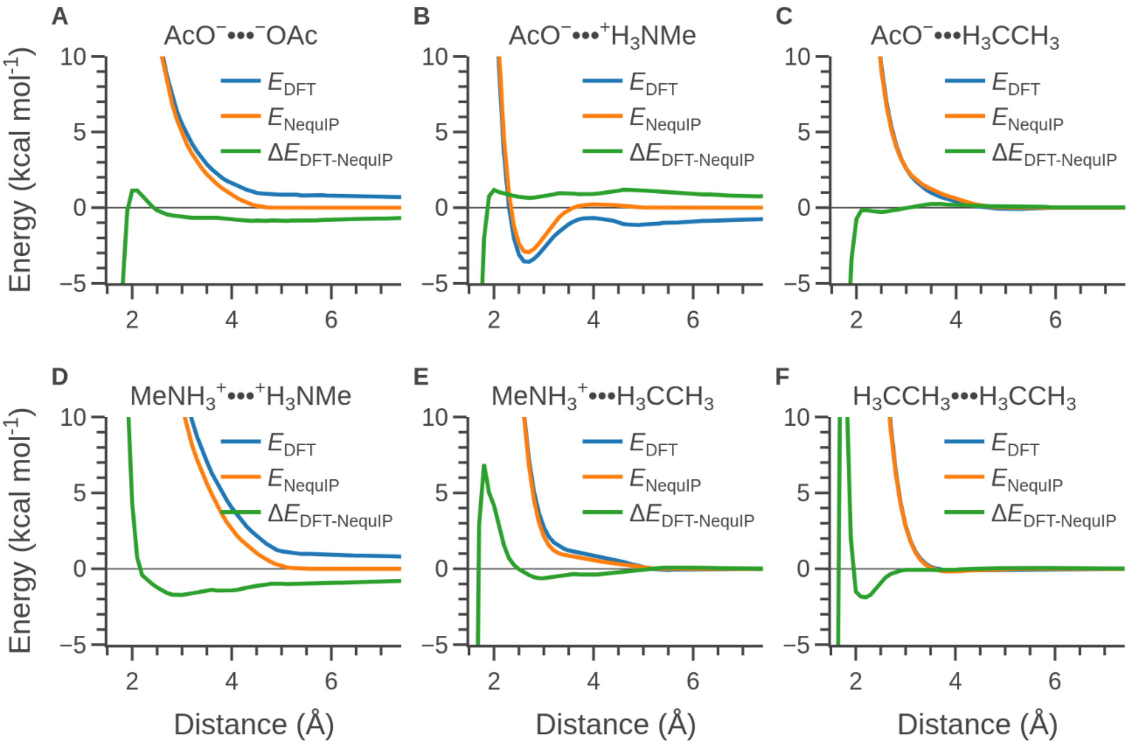

Figure S12. Interaction energies in systems composed of acetate, methylammonium, and ethane pairs calculated with DFT level (blue lines), NequIP (**T3_H_EF** model, orange lines), and their difference (green lines).

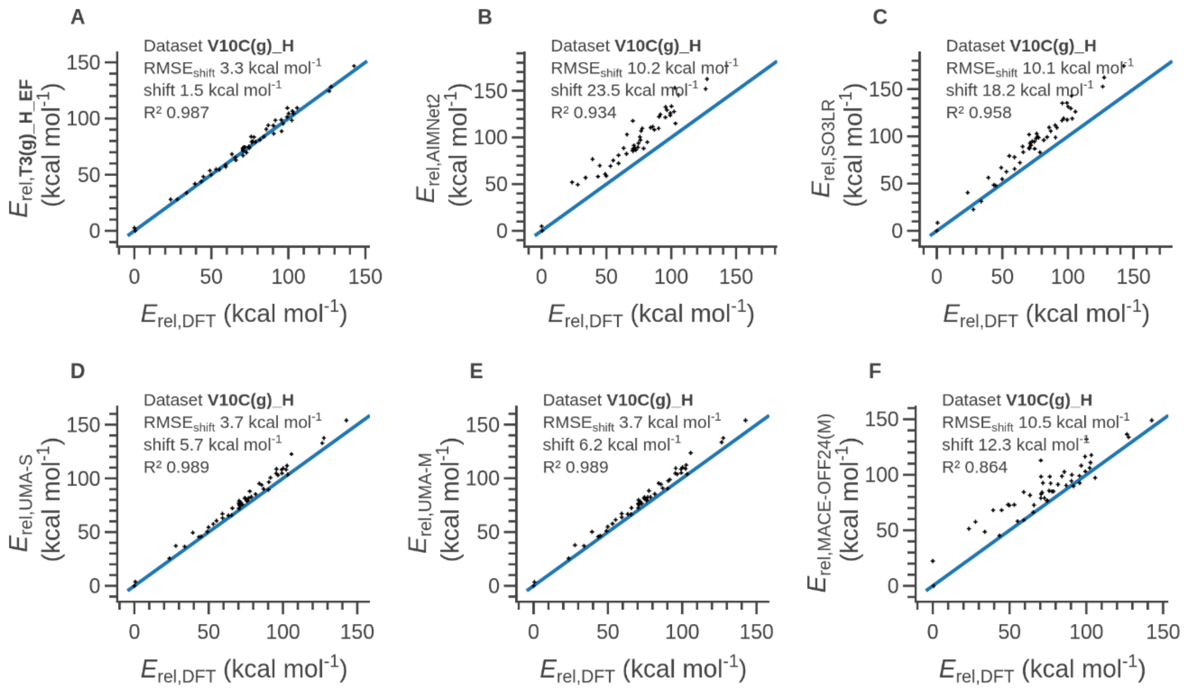

Figure S13 . Correlation between relative conformational energies at the DFT level ($E_{\mathrm{rel,DFT}}$) on **V10C(g)_H** dataset and relative conformational energies computed using (A) **T3(g)_H_EF** model, (B) AIMNet2, (C) SO3LR, (D) UMA-S, (E) UMA-M, and (F) MACE-OFF24(M) models. Note that models in panels B-F were *not* trained at the same DFT level as was used for the **V10C(g)_H** dataset.

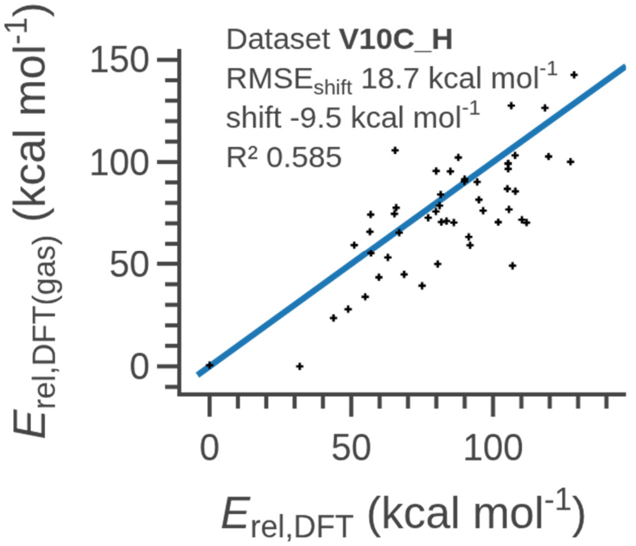

Figure S14 . Correlation between relative conformational energies at the DFT level ($E_{\mathrm{rel,DFT}}$) on **V10C_H** dataset and the relative conformational energies at the DFT(g) level at the same geometries (energies from the **V10C(g)_H** dataset).

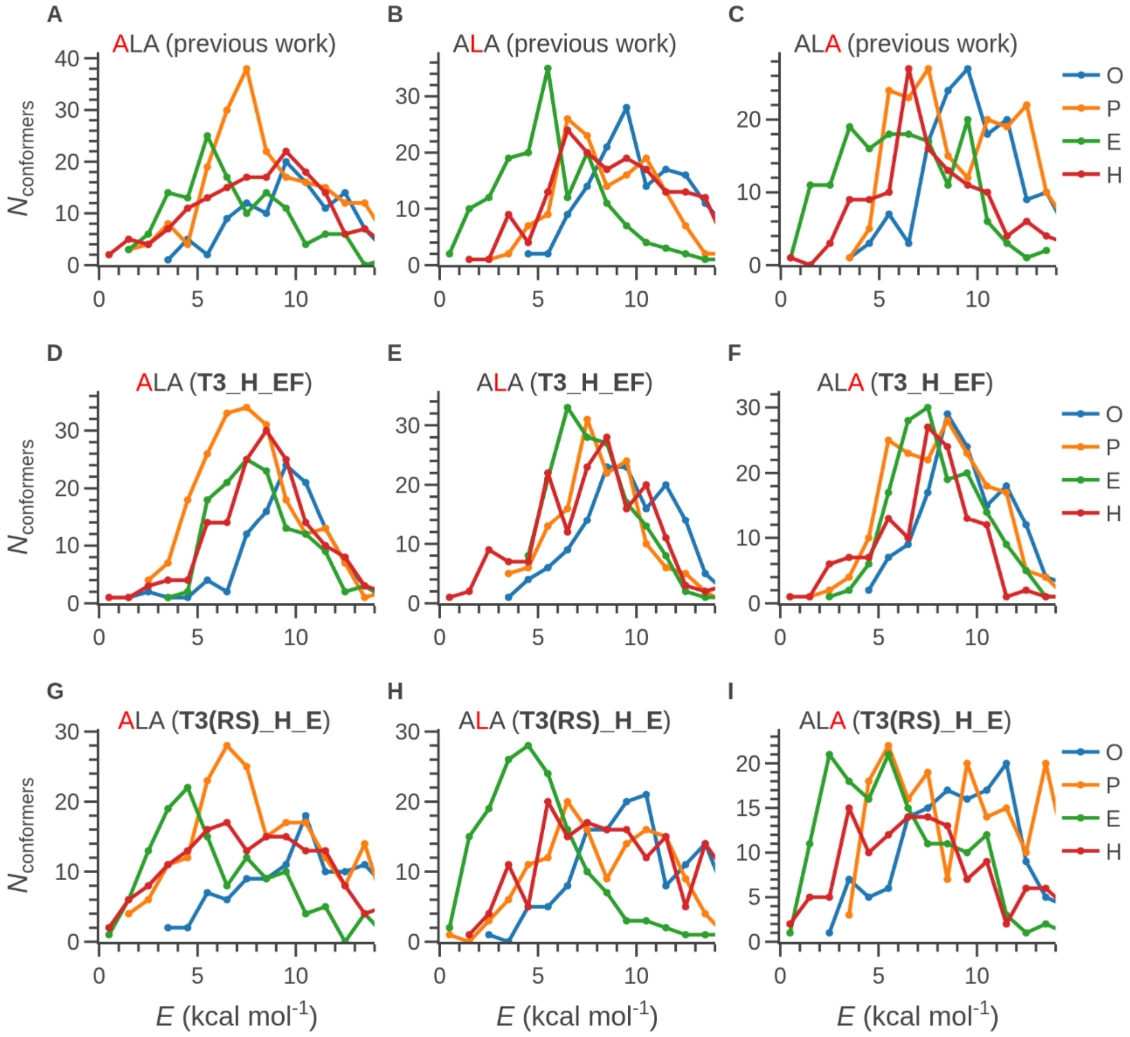

Figure S15. Energy histograms of ALA tripeptide conformers. Geometries were the same as used in Ref. 50 in the main text. (A-C) Energies from the Ref. 50 in the main text, calculated at BP86-D3BJ^{Rezac}/dgauss-dzvp + COSMO-RS (BP_TZVPD_FINE_21.ctd parameters) level in MeOH, (D-F) calculated by **T3_H_EF** model (trained at DFT level from this work), (G-I) calculated by **T3(RS)_H_E** model, which was trained on COSMO-RS level energies. The different structural types are indicated by line colors: α-helix (H; red), extended (E; green), polyproline II (P; orange); other (O; blue). Each column corresponds to different amino acid, which is also highlighted in red: (A, D, G) is the first residue, (B, E, H) is the second residue and (C, F, I) is the third residue.

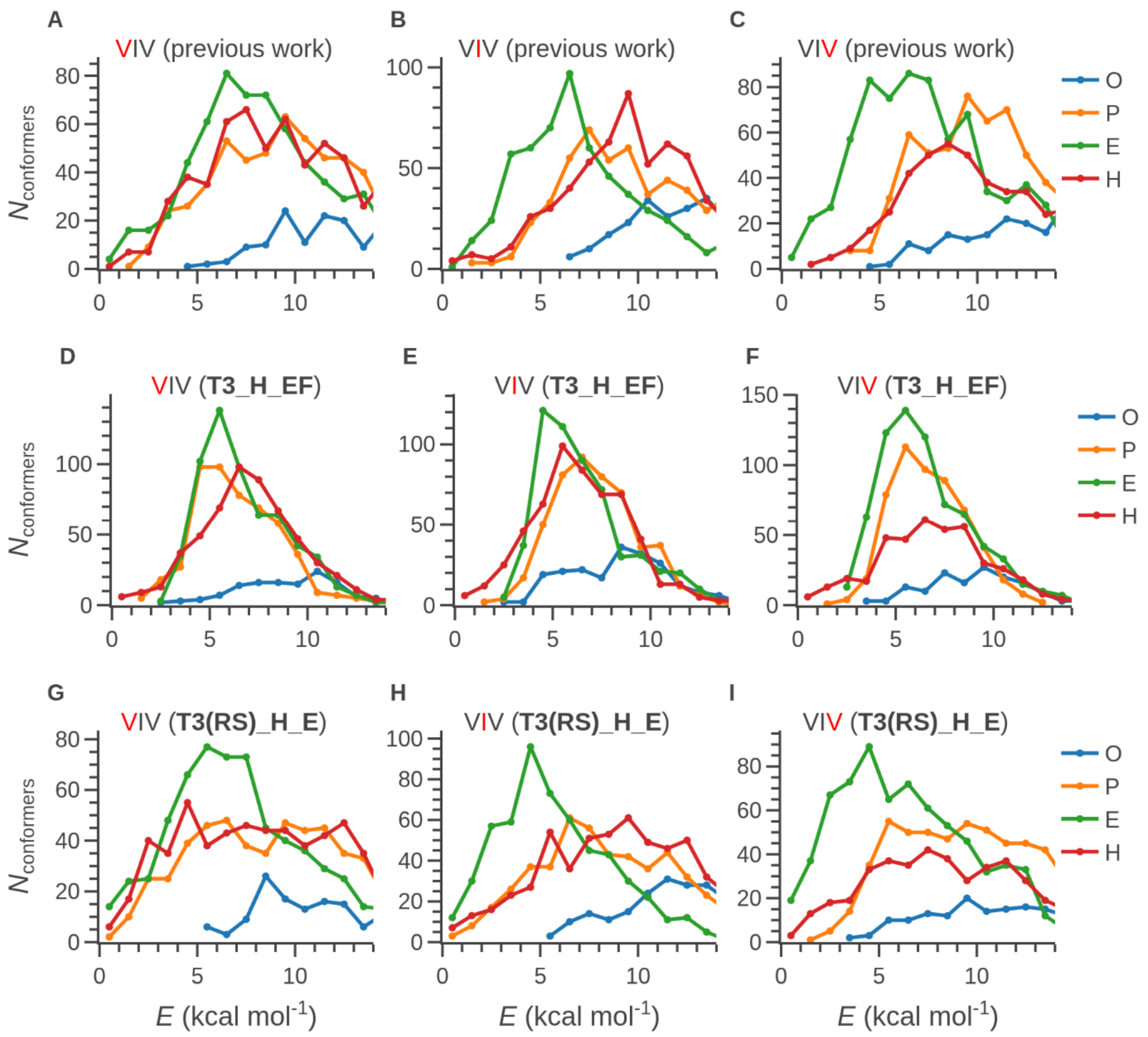

Figure S16. Energy histograms of VIV tripeptide conformers. See Figure S15 for description.

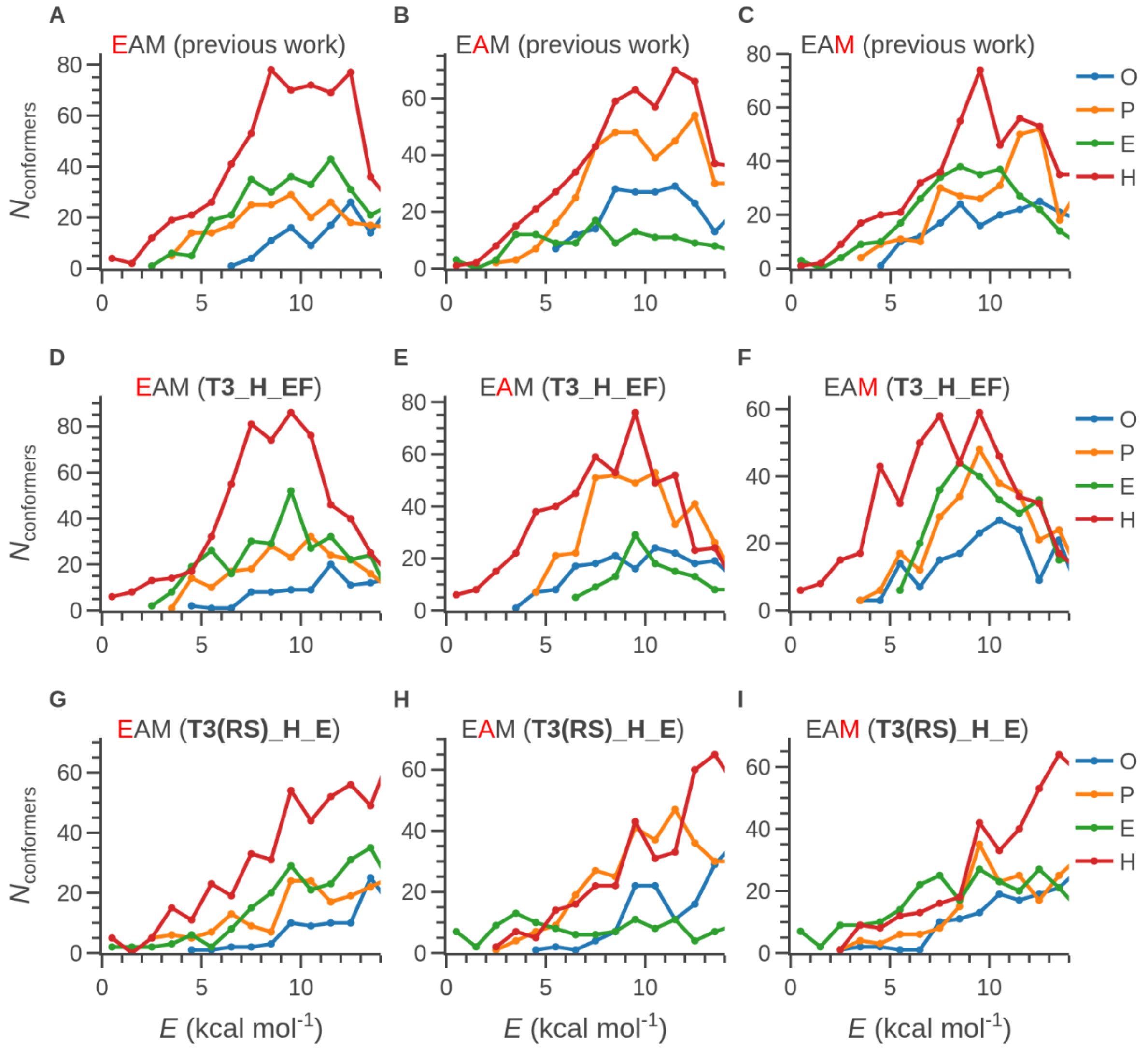

Figure S17. Energy histograms of EAM tripeptide conformers. See Figure S15 for description.

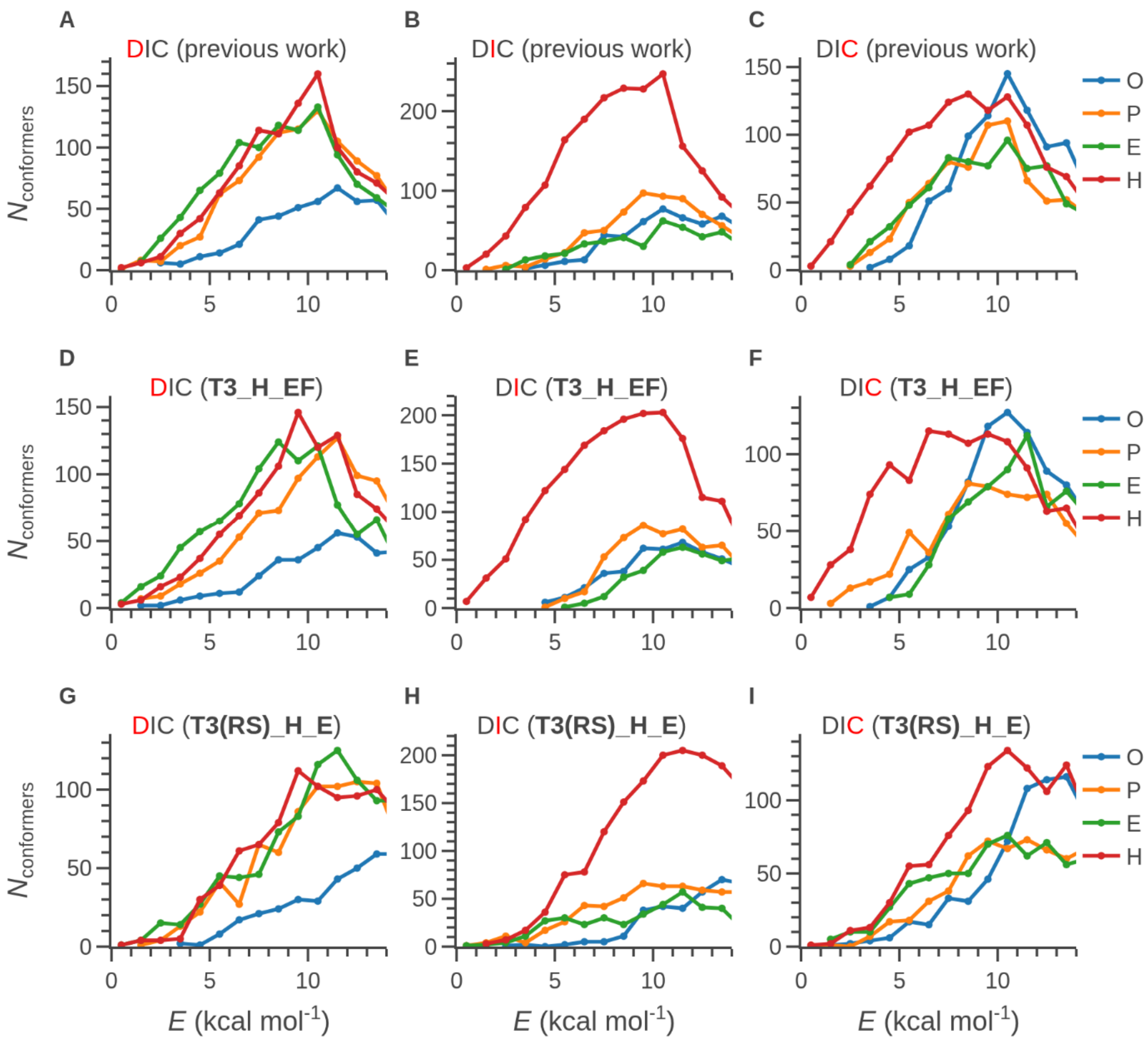

Figure S18. Energy histograms of DIC tripeptide conformers. See Figure S15 for description.
